# Prospects and Challenges of Proton Conducting Cerates in Electrochemical Hydrogen Devices for Clean Energy Systems: A Review

**DOI:** 10.1002/gch2.202500119

**Published:** 2025-06-09

**Authors:** M. Khalid Hossain, Ranjit C. Das, M. Imran Hossain, M. Atikur Rahman, Prabhu Paramasivam, Ripel Chakma, Mongi Amami, Mohamed H. H. Mahmoud, R. Bousbih, Rajesh Haldhar, Kenichi Hashizume

**Affiliations:** ^1^ Department of Advanced Energy Engineering Science Interdisciplinary Graduate School of Engineering Science Kyushu University Fukuoka 816‐8580 Japan; ^2^ Institute of Electronics Atomic Energy Research Establishment Bangladesh Atomic Energy Commission Dhaka 1349 Bangladesh; ^3^ Materials Science and Engineering Florida State University Tallahassee FL 32306 USA; ^4^ Institute for Micromanufacturing Louisiana Tech University Ruston LA 71270 USA; ^5^ Department of Electrical and Electronic Engineering University of Chittagong Chittagong 4331 Bangladesh; ^6^ Centre for Research Impact & Outcome Chitkara University Institute of Engineering and Technology Chitkara University Rajpura Punjab 140401 India; ^7^ Department of Mechanical Engineering Mattu University Mettu 318 Ethiopia; ^8^ Department of Electrical and Electronic Engineering Dhaka University of Engineering & Technology Gazipur 1707 Bangladesh; ^9^ Department of Chemistry College of Sciences King Khalid University P.O. Box 9004 Abha 62217 Saudi Arabia; ^10^ Department of Chemistry College of Science Taif University P.O. Box 11099 Taif 21944 Saudi Arabia; ^11^ Department of Physics Faculty of Science University of Tabuk Tabuk 71491 Saudi Arabia; ^12^ School of Chemical Engineering Yeungnam University Gyeongsan 38541 Republic of Korea

**Keywords:** cerate proton conductor, electrochemical hydrogen device, hydrogen isotope separator, hydrogen pump, hydrogen sensor, solid oxide electrolyte, tritium recovery and monitoring

## Abstract

The growing demand for green energy and global concern about environmental issues raise the demand for alternative, environmentally friendly energy sources. Electrochemical hydrogen devices are widely investigated as a potential solution for clean and renewable energy. Proton‐conducting oxides (PCOs) used as an electrolyte are required in electrochemical devices to transport protons. Chemical stability and proton conductivity are essential properties to evaluate a suitable electrolyte for these devices. Doped cerate‐based materials exhibit excellent proton conductivity and chemical stability, making them suitable as electrolyte materials for hydrogen devices. Techniques including doping, co‐doping, sintering aid, and different fabrication processes enhance the proton conductivity and mechanical stability of proton‐conducting materials. This paper highlights the current development of cerate‐based PCOs used as an electrolyte in electrochemical devices named hydrogen pumps, hydrogen isotope separation systems, tritium recovery systems, and hydrogen sensors, which could be used in the nuclear fusion reactors, among other electrochemical hydrogen devices. The center part of this review paper summarizes the most recent research studies on these applications and offers a thorough understanding of the impact of doping, different synthesis methods, sintering aids, and operating environments on the composition, morphology, and performance of cerate electrolyte materials. The challenges and prospects of proton‐conducting cerates are also discussed. This paper provides an insightful pathway for the researcher to further research in this field.

## Graphical abstract

This review paper highlights the current development of cerate‐based proton‐conducting oxides (PCOs) used as an electrolyte in electrochemical devices named hydrogen pumps, hydrogen isotope separation systems, tritium recovery systems, and hydrogen sensors. The challenges and prospects of proton‐conducting cerates are also discussed. Finally, this paper provides an insightful pathway for the researcher to further research in this field.

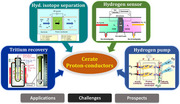

## 1 Introduction

The emission of greenhouse gases and other combustion pollutants negatively impacts our society and environment^[^ ^1^, ^2^, ^3^ ^]^ In addition, the depletion of energy sources and the growing energy demand have been the driving force behind the discovery of new energy resources and sustainable energy systems.^[^ ^4^, ^5^, ^6^, ^7^, ^8^, ^9^ ^]^ Therefore, the demand for green energy hydrogen is gradually increasing to ensure energy security and mitigate global air pollution. The electrochemical devices that use H^+^ as an electrolyte have been thoroughly investigated to meet the energy demand and create a sustainable energy system. Among different hydrogen device applications, Protonic ceramic fuel cells (PCFC) are utilized in the sustainable energy conversion industry, while hydrogen pumps are used to extract hydrogen from steam reforming.^[^ ^10^, ^11^, ^12^, ^13^, ^14^, ^15^ ^]^ Furthermore, the development of electrolyte materials significantly impacts the performance of hydrogen devices.^[^ ^16^ ^]^ As a result, developing a suitable material, synthesis processes, and evaluating the performance of the hydrogen devices have been the center of the research.

Proton‐conducting electrolytes offer several advantages over oxide‐ion conductors, such as high conductivity at intermediate temperatures, low activation energy, cost‐effectiveness, and long‐term stability.^[^ ^17^, ^18^, ^19^, ^20^, ^21^ ^]^ Proton conducting materials are used as electrolytes in a wide range of electrochemical devices, including hydrogen sensors,^[^ ^22^, ^23^, ^24^, ^25^ ^]^ humidity sensors,^[^ ^26^, ^27^, ^28^, ^29^, ^30^ ^]^ hydrogen pumps^[^ ^31^, ^32^, ^33^ ^]^ hydrogen gas separation systems,^[^ ^34^, ^35^, ^36^ ^]^ water electrolyzer membranes,^[^ ^37^, ^38^ ^]^ batteries,^[^ ^39^, ^40^, ^41^, ^42^, ^43^, ^44^, ^45^, ^46^ ^]^ and fuel cell electrolytes.^[^ ^47^, ^48^ ^]^ Solid‐state ion‐conducting ceramics called PCO exhibit significant proton conductivity at moderate temperatures. Iwahara et al. first published a paper on proton conductivity in oxides in the 1980s.^[^ ^49^, ^50^ ^]^ Kreuer reported the chemical and structural parameters that govern PCOs' proton transport characteristics and defect mechanism.^[^ ^51^ ^]^ Understanding proton generation and transportation gives us an idea to develop suitable PCOs.

PCOs are promising materials for energy conversion and storage device applications such as PCFC, ceramic fuel cells, membrane reactors, and ammonia synthesis.^[^ ^52^, ^53^, ^54^ ^]^ The Lattice defect oxides, i.e., perovskite proton conductor, have high conductivity and can be employed at a high temperature, typically 400–1000 °C. However, most PCOs, except for perovskite, reduce their ionic conductivity due to decomposition and dehydration.^[^ ^55^, ^56^, ^57^, ^58^, ^59^, ^60^, ^61^, ^62^ ^]^ Perovskite PCOs have excellent proton conductivity due to their significant negative enthalpies for hydration and low activation energies for proton migration.^[^ ^63^, ^64^ ^]^ Compared to other perovskites, cerate‐based PCOs exhibited promising protonic conductivity because they have low electron negativity, large lattice volume and distortion, and crystal structure.^[^ ^51^, ^65^ ^]^ BaCeO_3_ showed the highest proton conductivity than other PCOs due to their high chemical stability and grain boundary properties.^[^ ^49^, ^66^ ^]^ However, the thermal and chemical stability of BaCeO_3_ is low because of the binary oxide decomposition.^[^ ^67^ ^]^

On the other hand, barium zirconates BaZrO_3_ exhibited excellent stability in harsh environments, but the proton conductivity of BaZrO_3_ is poor.^[^ ^67^, ^68^, ^69^, ^70^ ^]^ An earlier study showed that chemical modification increases the stability of cerate‐based materials with high proton conductivity.^[^ ^51^ ^]^ Trivalent atoms have been partially doped with trivalent dopant cations, like yttrium ion (Y^3+^), to improve the proton conductivity and stability of cerate‐based proton conductors. Trivalent dopant cations are vital in developing oxide ion vacancies, which help to produce protons in the H^+^ atmosphere.^[^ ^67^ ^]^ The chemical stability, proton conductivities, hydrogen pumping abilities, tritium recovery efficiency, and hydrogen sensing capabilities of BaCeO_3_ and SrCeO_3_ make them ideal materials for fusion reactors. Thus, researchers are interested in lattice fault‐type oxides, which can be employed as solid electrolytes in electrochemical devices that run at intermediate and high temperatures.

Compared to the other perovskite proton conductors, the cerate‐based proton conductors are a potential candidate for developing highly reliable and fast‐response hydrogen sensors due to their high conductivity with increasing temperature and chemical stability under harsh process environments.^[^ ^71^ ^]^ Accurate measurement of tritium concentrations is essential in the fusion reactor's liquid blankets. Unlike its heavier isotope deuterium, readily available in water, tritium is a scarce isotope of hydrogen that needs to be bred within the reactor itself. Hydrogen isotope sensors can ensure the self‐sufficiency of tritium in breeding systems through reliable measurements. The inherent ability of the proton conductors to transport protons and oxygen ions extends the functionality of such hydrogen sensors to sense hydrogen isotopes effectively and efficiently in various compounds.^[^ ^72^ ^]^ Kawamura et al. reported an electrochemical hydrogen pump (EHP) using SrCe_0.95_Yb_0.05_O_3‐α_ (SCO) cerate‐based PCO for use in a fusion reactor's blanket tritium recovery system.^[^ ^73^ ^]^ The hydrogen transportation capability of the electrochemical device was improved by modifying the electrodes.^[^ ^74^ ^]^ The sputtering method attaches electrodes like Platinum and leads to the SrCe_0.95_Yb_0.05_O_3‐α_ sample. When the sputtered electrode was used, the H^+^ transport capability was improved. For example, at 0.1% H_2_ concentration, the current density increased multiple times more than the conventional Platinum electrode.^[^ ^75^ ^]^ However, the lower protonic conductivity of ceramics such as SrCe_0.95_Yb_0.05_O_3‐α_ is a concern, and it may result in less efficient fuel cells than those based on polymer electrolytes. The poor catalytic activity on the anode side would contribute to incomplete CH_4_ to H_2_ conversion, resulting in inferior cell performance compared to hydrogen‐fed fuel cells.^[^ ^76^ ^]^

BaCe_0.8_Y_0.2_O_3−δ_ (BCY), the most investigated proton‐conducting material, is commonly used as a dense membrane for hydrogen separation. Nevertheless, the exceptionally high sintering temperature (>1500 °C) presents a barrier to the fabrication of dense BaCe_0.8_Y_0.2_O_3−δ_ membranes. The high‐temperature proton‐conducting ceramic membranes and Ni‐based metallic conductors have drawn the attention of researchers. The transport proton of the hydrogen separation membrane offers faster electron conduction and surface exchange kinetics with mechanical stability.^[^ ^77^ ^]^ However, the hydrogen separation of cermet membranes is primarily restricted by ceramic proton‐conducting phases since nickel has a relatively high electronic conductivity. The cerate‐zirconate doped materials, for example, BaZr_0.1_Ce_0.7_Y_0.2_O_3−δ_ (BZCY), showed higher proton conductivity and excellent thermal and chemical stability in harsh environments (containing water and CO_2_).^[^ ^78^, ^79^ ^]^ Ni‐BZCY has since become one of the most extensively investigated cermet for hydrogen permeation membranes.^[^ ^33^, ^80^, ^81^, ^82^ ^]^ In hydrogen pumps, the cerate‐based materials SrCe_0.95_Yb_0.05_O_3‐α_ represent outstanding performance in terms of proton conduction than other PCOs.^[^ ^83^ ^]^ Cerate‐based proton conductors demonstrate high protonic conductivity and low activation energy, making them useful for solid oxide fuel cells and electrolysis operations.^[^ ^84^, ^85^ ^]^ However, the limitation of the cerate‐based proton conductor is low current efficiency at high current density, durability, and stability. Irshad et al. synthesized (BaCe_0.97_M_0.03_O_3‐δ_) with improved microstructures compared to traditional chemical methods. The material made with lemon juice even achieved a promising power density, suggesting its potential for efficient and sustainable fuel cell applications.^[^ ^86^ ^]^ The durability and stability of hydrogen pumps can be increased by doping materials like Zr in cerate‐based materials. Babar et al. explore copper and zirconium doping to improve conductivity in a fuel cell electrolyte. Doping with 2% copper in BaCe_0.7_Zr_0.1_Dy_0.2‐x_O_3‐δ_ (BCZD) reduces sintering temperature and increases conductivity, while also maintaining stability.^[^ ^87^ ^]^ The current efficiency can be enhanced in hydrogen pumps by increasing the water vapor supply in the cathode.^[^ ^88^ ^]^

This review aims to give a thorough understanding and idea of proton‐conductive cerate‐based materials to provide a guideline for developing new cerate materials with high stability and proton conductivity. This reviewed paper also gives an overview of proton‐conducting cerate perovskite materials and brief details of hydrogen devices in the first section. In the following part, this review paper summarizes the application of cerate‐based proton‐conducting materials as an electrolyte in the electrochemical hydrogen devices, which could be used in the nuclear fusion reactors, among other electrochemical hydrogen devices. Additionally, it presents the recent development and potential of cerate‐based perovskite proton‐conducting material for hydrogen sensors, tritium recovery systems, hydrogen isotope separation, and hydrogen pumps. Finally, this paper also discusses the challenges and prospects of cerate‐based perovskite materials based on the published literature and the author's experience in proton‐conducting cerates for hydrogen devices.

## 2 Proton‐Conducting Cerates and Cerate‐Based Electrochemical Hydrogen Devices

### 2.1 Proton‐Conducting Cerates

**Figure 1:**
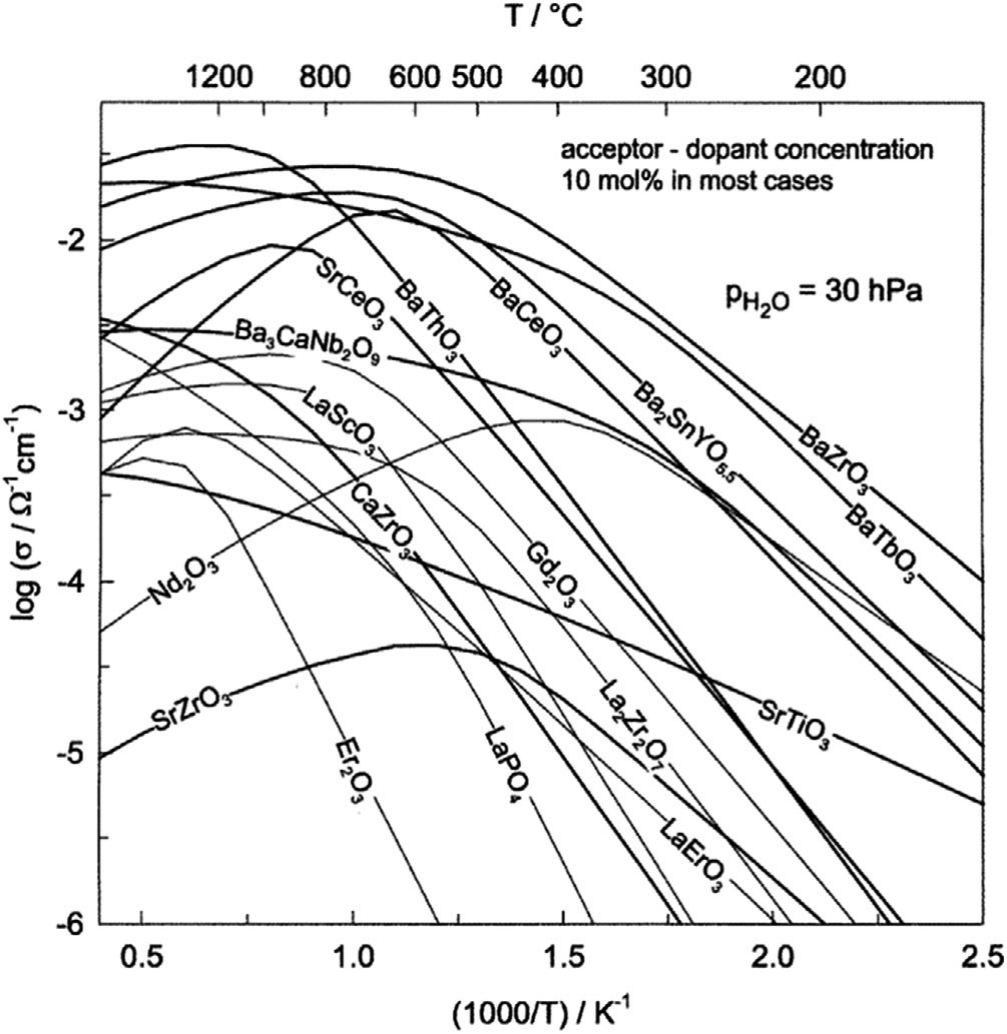
A graph of proton conductivity for different oxides is constructed based on data on proton concentrations and mobilities. Bold lines indicate the conductivity of perovskite‐type oxides. Reproduced with permission from ref. [51].

Cerate‐based perovskite proton conductors, such as BaCeO_3_ and SrCeO_3_, exhibited high proton conductivity due to their crystal structure, high lattice volume and distortion, and low electronegativity. Different strategies, such as doping with different materials, were developed to improve the performance and properties of cerate‐based proton conductors. Among all cerate‐based proton conductors, BaCeO_3_ and SrCeO_3_ were widely studied due to their good proton conductivity and stability in the harsh atmosphere (water vapor, CO_2_) at intermediate temperatures.^[^ ^67^ ^]^ BaCeO_3_ showed the highest proton conductivity compared to the other cerate proton conductors due to their largest ionic radius cation, large lattice parameter, low grain‐boundary resistance, and low electronegativity.^[^ ^89^, ^90^ ^]^ **Figure** **1** represents calculated proton conductivities of different oxides using the information on proton concentrations and mobilities. Compared to the other perovskite cerate materials, BaZrO_3_ and BaCeO_3_ materials are the best proton conductor materials (Figure 1).^[^ ^51^ ^]^ However, BaCeO_3_ has low chemical stability that prevents it from being used as an electrolyte in the application of electrochemical devices.

**Figure 2:**
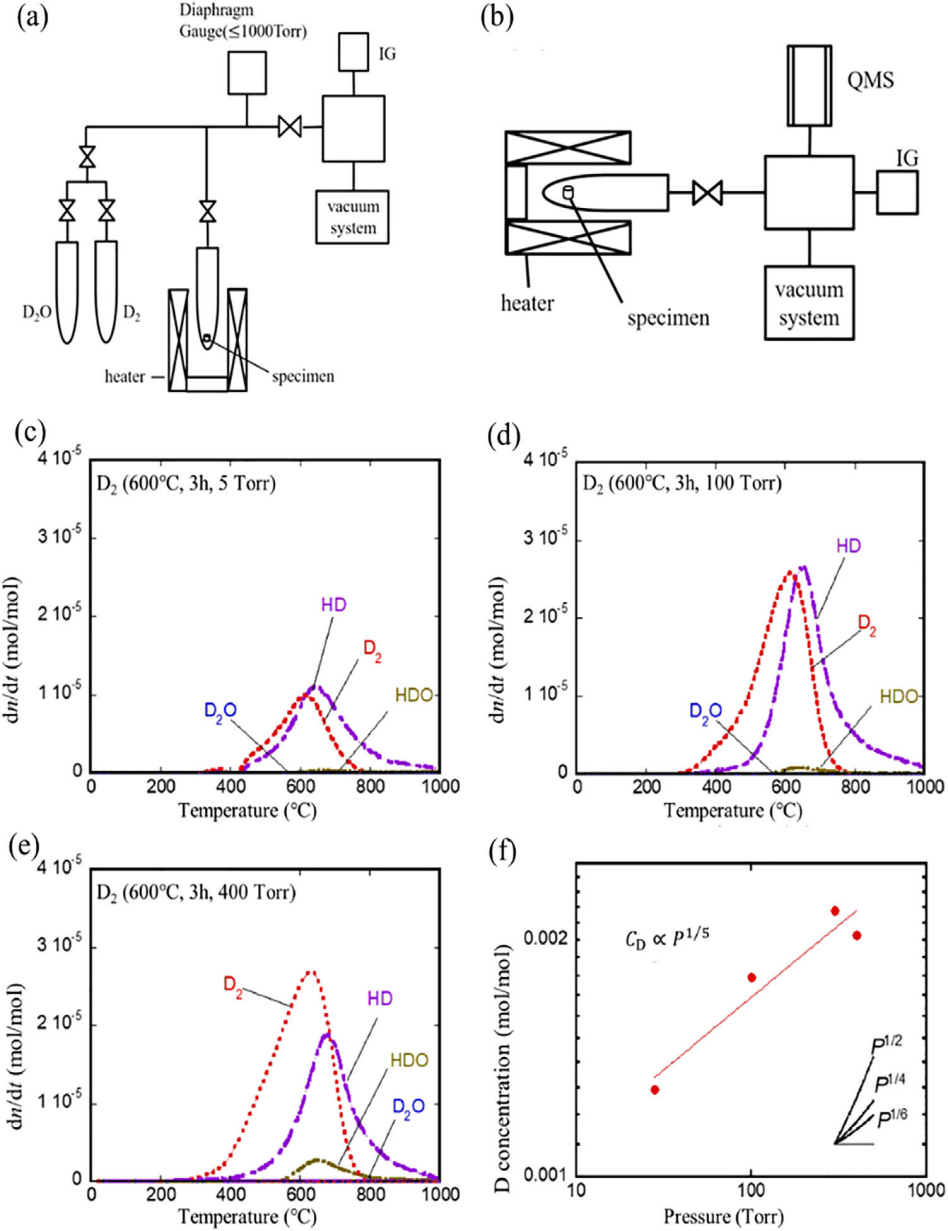
a,b) An illustration of the equipment used in the experiments for (a) hydrogen absorption and (b) desorption. The following graphs show the hydrogen release behavior for a sample exposed to D_2_: c) exposure pressures of 5 Torr, d) 100 Torr, e) 400 Torr, and f) exposure pressure against dissolved hydrogen concentration curve. Reproduced with permission from ref. [91].

Recent research has shown that the proton conductivity of BaCeO_3_ can be enhanced by doping with trivalent rare earth material and zirconate materials. Hossain et al. exposed deuterium gas (D_2_) and heavy water vapor (D_2_O) at 873 or 973 K for 5 h to study the dissolution and release behavior of hydrogen in Y‐doped BaCeO_3_ (BaCe_0.9_Y_0.1_O_3_) proton conductor, shown in **Figure** **2**.^[^ ^91^ ^]^ In Figure 2c–f, they investigated how much hydrogen isotopes (deuterium) dissolve in a material at different gas pressures. It was found that the amount of absorbed hydrogen increased with pressure, but not as strongly as predicted by Sieverts' Law (which predicts a square root dependence). This suggests that the pressure affects the number of sites available for hydrogen storage within the material. Furthermore, the type of gas released upon heating the material depended on the exposure pressure. At lower pressures, more HD gas (containing one hydrogen and one deuterium atom) was released, while higher pressures favored D_2_ gas (pure deuterium). This indicates that higher pressure promotes the exchange of lighter hydrogen atoms in the material with the incoming deuterium gas. Gu et al. showed that BaCeO_3_ doped with rare earth elements such as samarium (Sm) and Gadolinium (Gd) showed higher proton conductivity compared to the other proton conductors.^[^ ^92^ ^]^ However, SrCeO_3_ proton conductors exhibited lower proton conductivity than BaCeO_3_ in reducing environments at high temperatures.^[^ ^67^ ^]^ For example, Iwahara et al. reported that at a temperature (800 °C), the proton conductivity of SrCeO_3_ was 10^−3^–10^−2^ S cm^−1^. However, SrCeO_3_ has higher chemical stability than BaCeO_3_. Some studies were conducted to improve the chemical stability of BaCeO_3_, where BaCeO_3_ was doped with zirconium (Zr), Niobium (Nb), Tin (Sn), and Titanium (Ti).^[^ ^67^, ^93^, ^94^ ^]^ This type of reported study showed that this approach reduced the proton conductivity of BaCeO_3_. Therefore, finding a suitable path to improve the ionic conductivity and stability of cerate‐based proton conductors is the research center.

**Table** **1** represents the performance of various electrolyte materials with their conductivity, chemical stability, operating temperature, sintering temperature, and synthesis methods.^[^ ^124^ ^]^ The performance includes the open‐circuit voltage (V) and peak power density (mW cm^−^^2^) of the various cerate‐based materials. Table 1 demonstrates that various electrolyte materials provide better conductivity and peak power density based on their synthesis method, operating temperature, and sintering temperature. It is noted that BaZr_0.8_X_0.2_O_3‐δ_ provides a peak power density of up to 340 mW cm^−2^ under 650 °C operating temperature, and SrCe_0.8_Y_0.2_O_3‐δ_ provides conductivity up to 12 × 10^−3^ S cm^−1^ under 1500 °C sintering temperature. **Table** **2** represents the performance of different hydrogen devices using various cerate‐based electrolytes, summarizing the conductivity, peak power density, and key findings of different materials under various operating temperatures and H_2_ Environmental Conditions. It is noted that fuel cells using various cerate‐based electrolyte materials provide outstanding performance, such as better conductivity and peak power density under different operating temperatures.

**Table 1:** Performance (peak power density, Pmax, and open‐circuit voltage, OCV) of different electrolyte materials with their conductivity and chemical stability.

Electrolyte materials	Synthesis method	Operating temperature [°C]	Sintering temperature [°C]	Conductivity, σ [S cm^−1^]	Chemical Stability in CO₂/H₂O Atmospheres	Open‐circuit voltage, OCV [V]	Peak power density, P_max_ [mW cm^−2^]	Refs.
BaZr_0.8_Y_0.15_Sm_0.05_O_3‐δ_	Citrate‐nitrate combustion	700	1600	–	Stable	1.01	180	[95]
BaZr_0.8_Y_0.15_Ca_0.05_O_3‐δ_	citrate‐nitrate combustion	700	1600	0.8 × 10^−3^ (in wet H_2_)	Stable	0.928	218	[96]
BaZr_0.8_Y_0.2_O_3‐δ_	EDTA and citric acid	700	1300	–	–	0.8	322.23	[97]
BaZr_0.8_X_0.2_O_3‐δ_	Citric acid	650	1150	2.2 × 10^−3^	–	0.88	340	[98]
BaZr_0.8_Y_0.2_O_3‐δ_	Combined EDTA‐citric acid	600	1450	–	Stable	0.6	198	[99]
Ba_0.5_Sr_0.5_Zr_0.5_Zn_0.5_O_3_	EDTA	–	1350	–	–	–	–	[100]
BaCe_0.6_Zr_0.3_Y_0.1_O_3‐δ_	Solid‐state reaction	500	1700	2 × 10^−3^	–	–	–	[101]
BaCe_0.65_Zr_0.20_Y_0.15_O_3‐δ_	Sol‐gel	700	1450	10.2 × 10^−3^	–	–	–	[102]
BaCe_0.9_Y_0.1_O_3‐δ_	Acetate‐H_2_O_2_ nitrate‐free combustion	‹400	1450	‐	–	–	–	[103]
BaCe_0.7_Zr_0.1_Y_0.2_O_3‐δ_	Acetate‐H_2_O_2_ combustion	850	1550	–	Stable	–	–	[104]
BaCe_0.65_Zr_0.2_Y_0.15_O_3‐δ_	SSR	800	1500	–	–	–	–	[105]
BaZr_0.8_Y_0.2_O_3‐δ_	Sol‐gel	500	1200	3.9 × 10^−4^	Stable	–	–	[106]
BaSn_x_Ce_0.8‐x_Yb_0.2_O_3‐δ_	‐	600	–	–	Stable	1.08	157	[107]
SrCe_0.8_Y_0.2_O_3‐δ_	Citrate‐nitrate combustion	–	1500	12 × 10^−3^	Stable	–	–	[108]
BaCe_0.8_Y_0.2_O_3‐δ_	Sol‐gel	600	1500	5.8 × 10^−3^	–	–	–	[109]
BaCe_0.7_Zr_0.1_Y_0.1_Yb_0.1_O_3‐δ_	Solid state reaction	600	1400	‐	–	–	–	[110]
Ba_0.95_Ce_0.8_Y_0.2_O_3‐δ_	Ethylene glycol and citric acid	550	1200	11 × 10^−3^	–	–	–	[111]
BaSn_0.7_In_0.15_Y_0.15_O_3‐δ_	Citrate‐nitrate combustion	700	1450	1.84 × 10^−3^	–	–	–	[112]
BaCe0.9Zr_0.2_Y_0.1_O_2.95_	SSRS	500	1700	8 × 10^−3^	–	–	–	[113]

**Table 2:** Performance of different hydrogen devices using cerate‐based electrolytes.

Device type	Electrolyte composition	Operating temperature [C]	H_2_ environmental condition	Conductivity, σ [S cm^−1^]	Peak power density, P_max_ [mW cm^−2^]	Key features	Refs.
PCFC	BaCe_0.5_Zr_0.35_Y_0.15_O_3‐δ_	600	Humidified H₂	–	635	Thin 2 µm electrolyte layer, gradient anode improves adhesion and OCV > 1 V	[114]
PCFC	BaZr_0.1_Ce_0.7_Y_0.2_O_3‐δ_	650	Humidified 50H_2_/50 N_2_	–	–	For pumping pure hydrogen, a Faradaic efficiency of 90% was achieved at 1.33 A cm^−2^, yielding a hydrogen flux of 8.7 mL⋅cm^−2^ ⋅min^−1^.	[115]
SOEC	BaCe_0.7_Zr_0.1_Y_0.16_Zn_0.04_O_3‐δ_	700	Dry 5% H_2_ in Ar and 3% wet Ar	3 × 10^−2^	–	The ASR value related to the second phase increased 144% in ≈70 h testing period.	[116]
SOFC	BaCo_0.4_Fe_0.4_Zr_0.2_O3‐δ	600	Humidified H₂	–	470	The findings demonstrated better electrochemical reaction at the TPB and improved gas diffusion.	[117]
PCFC	BaCe_0.7_Zr_0.1_Y_0.1_Yb_0.1_O_3‐δ_	600	Air/H2 gradient	3.7 × 10^−3^	121	The results demonstrated P_max_ of 97–121 mW cm^−2^ at 600–650 °C under an air/H_2_ gradient, with OCVs of 0.94–0.97 V.	[118]
PCFC	BaCe_0.7_Zr_0.1_Y_0.15_Zn_0.05_O_3−δ_	700	Humidified H₂	–	872	The results demonstrated the material's high density and good proton conductivity at moderate temperatures (400–700 °C).	[119]
PCFC	BaZr_0.1_Ce_0.7_Y_0.2_O_3‐δ_	700	Humid air and H_2_	–	360	Findings indicate that after being sintered with NiO aid at 1400 °C for 6 h, the material can be densified.	[79]
PCFC	BaCe_0.4_Zr_0.4_Y_0.1_Yb_0.1_O_3‐δ_	600	H_2_ and CH_4_	–	690	The electrolyte shows superior long‐term stack durability.	[120]
SOFC	BaCe_0.36_Fe_0.64_O_3–δ_	700	Wet H_2_	–	1525	The BCF36 cathode microstructure is modified by spraying, resulting in a record cell performance of 1525 mW cm^−2^ at 700 °C.	[121]
PCFC	BaCe_0.7_Zr0.1Y_0.2_O_3‐δ_	600	Humidified H₂	6.35 × 10^−3^	465	The operating power density significantly drops during the first 10 h of the 30‐h test before stabilizing at 300 mW cm^−2^.	[122]
PCFC	BaZr_0.1_Ce_0.7_Y_0.2_O_3‐δ_	600	H_2_	–	391	At 600 °C, an anode‐supported PCFC with an LSCF2.7 cathode and BZCY172 electrolyte provides better durability and an improved peak power o/p.	[123]

### 2.2 Cerate Material‐Based Different Types of Electrochemical Hydrogen Devices

Cerate material‐based electrochemical hydrogen devices can be classified as hydrogen pumps, hydrogen isotope separation systems, tritium recovery systems, and hydrogen sensors, which are briefly defined below.

**Hydrogen pump**: Hydrogen pumps based on cerate materials are electrochemical devices that use proton‐conducting ceramics to transport hydrogen through solid‐state membranes at high temperatures. Hydrogen splits into protons and electrons at the anode when an electric potential is applied across the ceramic membrane in these devices. Protons migrate through the ceramic electrolyte, and electrons travel through an external circuit, finally joining again to generate hydrogen gas at the cathode.^[^ ^125^ ^]^ These pumps' efficiency is greatly impacted by the properties of the ceramic materials that are used. For instance, doping SrCeO₃ with ytterbium improves its stability and proton conductivity, which makes it more suitable for high‐temperature applications. It has been demonstrated that these modifications enhance the performance of hydrogen pumps, especially in situations where effective hydrogen transport is essential, such as tritium recovery systems in fusion reactors.^[^ ^74^ ^]^

**Hydrogen isotope separation systems**: Hydrogen isotope separation systems are a form of electrochemical hydrogen device that uses ceramic membranes that conduct protons, usually perovskite‐type oxides like BaZrO₃ or BaCeO₃, to transport hydrogen isotopes selectively when an electric field is applied. These devices separate hydrogen isotopes (tritium, deuterium, and protium) through the exploitation of the differential mobility inside the ceramic lattice.^[^ ^126^ ^]^ In such systems, hydrogen ions (protons) are driven through the material by applying a voltage across the ceramic membrane. They can separate because lighter isotopes, like protium, migrate more easily than heavier ones, like deuterium or tritium, due to variations in mass and contact with the lattice. Compared to conventional separation methods, this approach has the potential for continuous operation and uses less energy.^[^ ^127^ ^]^

**Tritium recovery systems**: Tritium recovery systems are electrochemical hydrogen devices that use proton‐conducting ceramics, especially doped zirconates like CaZr₀.₉In₀.₁O₃₋α and SrZr₀.₉Yb₀.₁O₃₋α, to extract tritium and other hydrogen isotopes from gas mixtures or water vapor in fusion reactor environments. These systems operate by applying an electric voltage over a ceramic membrane, which forces protons across it and makes it possible to separate and recover hydrogen isotopes.^[^ ^56^ ^]^ A key component of these systems is the electrochemical hydrogen pump, which utilizes the strong proton conductivity and chemical stability of perovskite‐type ceramics at high temperatures (usually between 873 and 1073 K). For instance, studies have demonstrated that using CaZr_₀.₉_In_₀.₁_O_₃₋α_ as the proton‐conducting membrane enables effective hydrogen extraction from helium‐hydrogen mixtures, with hydrogen recovery efficiency exceeding 99% in certain conditions.^[^ ^128^ ^]^

**Hydrogen sensors**: Hydrogen sensors refer to sensors that use cerate‐based materials as the primary sensing component to detect hydrogen gas. Based on electrochemical principles, these sensors measure the concentration of hydrogen by producing a quantifiable electrical signal from the interaction between the hydrogen gas and the cerate material. The hydrogen gas usually reacts with the material during the electrochemical process, changing its conductivity or potential, which can be measured with electrodes.^[^ ^129^ ^]^ Hydrogen sensors based on cerate operate over a broad temperature range, usually between room temperature and 500 °C. They may be used in both ambient and high‐temperature industrial settings because of their broad operating temperature range.^[^ ^130^ ^]^ Cerate‐based hydrogen sensors tend to exhibit long‐term stability due to the robust nature of cerate materials, which resist degradation over time. This makes them suitable for continuous monitoring in critical applications.^[^ ^131^ ^]^

## 3 Operating Principle or Mechanism of Proton‐Conductor‐Based Electrochemical Hydrogen Devices

Electrochemical hydrogen devices consist of three fundamental elements: that are electrode (anode), proton‐conducting electrolyte, and the electrode (cathode).^[^ ^132^, ^133^ ^]^ PCOs have long been used in electrochemical device applications due to the proton's (H^+^) small radius, which allows for greater intercalation into the cathode's layered structure.^[^ ^134^, ^135^ ^]^ Proton‐conducting electrolytes have two principal functions: Electromotive force (EMF) and electrochemical hydrogen transport in solids that can be used in electrochemical hydrogen devices.

The function of EMF is based on the principle of the galvanic cell that uses a proton conductor as a solid electrolyte in electrochemical hydrogen devices.^[^ ^136^ ^]^ The electrochemical device is called a hydrogen sensor when EMF is used to generate a signal. In contrast, electrochemical hydrogen transportation in solids based on the migration of protons is used in hydrogen pumps, where hydrogen extraction or separation happens to remove hydrogen from a gas mixture. A similar method is being used to separate hydrogen isotope and recover tritium (^3^H) in the tritium recovery system and hydrogen isotope separation system.^[^ ^137^, ^138^ ^]^

### 3.1 Mechanism of Hydrogen Pumps

**Figure 3:**
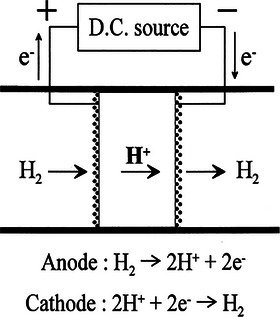
A schematic diagram of the hydrogen pump. Reproduced with permission from ref. [142].

Numerous studies have been conducted to develop a technology for producing pure hydrogen, including pressure swing adsorption, cryogenic distillation, and nongalvanic hydrogen separation pumps.^[^ ^139^, ^140^, ^141^ ^]^ An EHP can be constructed by using proton conductors. The principal operation and construction of the EHP are represented in **Figure** **3**.^[^ ^142^ ^]^ The system consists of **two electrodes (anode and cathode)** separated by a **proton‐conducting electrolyte membrane**. When the direct current is applied to the proton‐conducting electrolyte, hydrogen is ionized at the anode to produce protons in the electrolyte.^[^ ^71^ ^]^ This reaction produces **protons (H⁺)** and **electrons (e⁻)**. A proton moves toward the cathode through the electrolyte layer, which converts it to hydrogen gas.^[^ ^143^ ^]^ This method uses separated hydrogen in a controlled way by the applied current in the electrolyte.

Hydrogen extraction and production have been achieved utilizing an EHP based on the proton conductor, for example, SrCe_0.95_Yb_0.05_O_3‐α_, BaCe_0.9_Nd_0.10_O_3−α_, CaZr_0.96_In_0.04_O_3_, and BaZr_0⋅1_Ce_0⋅7_Y_0⋅2_O_3‐δ_.^[^ ^140^, ^144^ ^]^ The protonic conductor BaZr_0⋅1_Ce_0⋅7_Y_0⋅2_O_3‐δ_ has excellent chemical stability at high humidity and high cerate protonic conductivities.^[^ ^140^ ^]^ Cerium is easily reduced to its +3 and +4 oxidation states by redox processes. This redox chemistry is especially important for processes involving hydrogen since materials containing cerium can operate as redox catalysts or redox‐active species. The stability and conductivity of cerate‐based PCOs at high temperatures are beneficial. This makes it possible for hydrogen pumps to function at higher temperatures when traditional proton exchange membrane (PEM) technologies might not be as effective. The ceramics proton conductor SrCe_0.95_Yb_0.05_O_3‐α_, BaCe_0.9_Nd_0.10_O_3−α_, and CaZr_0.96_In_0.04_O_3_ are used to enhance the ion conductibility, sinterability, and material's chemical stability.^[^ ^145^, ^146^ ^]^ The ceramic composition becomes the purest protonic conductor at high temperatures for exposing hydrogen gas. Alternatively, at high temperatures, cerium oxide can be reduced by hydrogen gas to generate cerium hydride (CeH_2_) or lower oxidation state cerium species. Because of the reversibility of this reduction process, cerium oxide can be renewed by reoxidation in environments with oxygen. However, a hydrogen pumping system can be manipulated by temperature, hydrogen pressure, and water mixture.^[^ ^140^ ^]^

Cerate‐based electrochemical hydrogen pumps have shown promising performances in applications involving the separation and purification of hydrogen, especially those that use doped barium cerate (BaCeO₃) materials. For instance, BaCe₀.₂Zr₀.₇Y₀.₁O₃_−δ_ (BCZY271)‐based proton‐conducting solid oxide cells (P‐SOCs) have been developed and are capable of working efficiently at intermediate temperatures (350–450 °C) with current densities ranging from 150 to 525 mA cm^−2^ at 1 V. These P‐SOCs have benefits in thermodynamic efficiency and material stability, which makes them suitable for a variety of hydrogen‐related applications.^[^ ^147^, ^148^ ^]^ Proton conductor electrolytes based on yttrium‐doped barium cerate, as BaCe₁–_x_Y_x_O₃_−δ_ (where *x* = 0.15), have also demonstrated improved proton conductivity in humidified conditions. According to studies, these materials' conductivity is greatly increased in moist air conditions, highlighting the role that water vapor plays in promoting proton conduction.^[^ ^149^ ^]^ Although their performance is affected by factors including operating temperature, humidity, and material composition, cerate‐based materials usually demonstrate excellent electrochemical properties and high proton conductivity.^[^ ^150^ ^]^

### 3.2 Mechanism of Hydrogen Isotope Separation Systems

**Figure 4:**
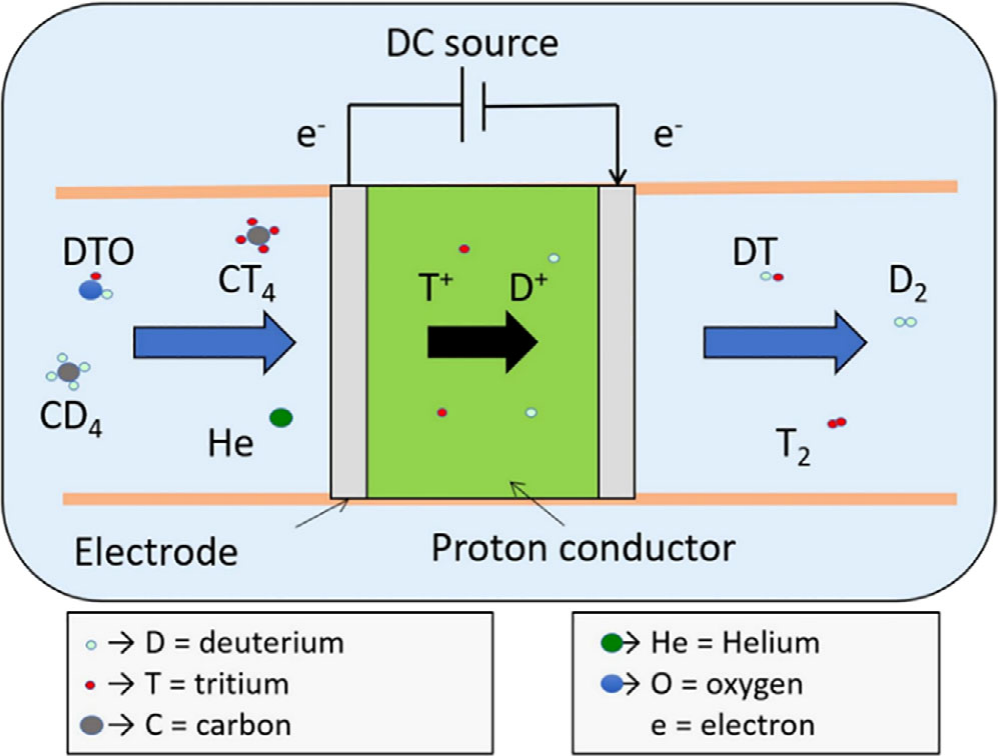
Diagram of the D‐T purification system, which uses isotope separators made of proton‐conducting oxide materials. Reproduced with permission from ref. [143].

In cerate‐based electrochemical hydrogen devices, hydrogen isotope separation primarily relies on the difference in ion mobility and diffusion rates arising from the mass disparity between isotopes. This mechanism enables the selective transport of protons (H⁺) and deuterons (D⁺) across a proton‐conducting ceramic membrane, typically composed of doped barium cerate.^[^ ^71^ ^]^ These materials demonstrate high proton conductivity at high temperatures (usually 500–800 °C).^[^ ^151^ ^]^ When a voltage is applied across the electrochemical cell, hydrogen isotopes from a gas mixture (e.g., H₂ and D₂ or H₂O and D₂O) are ionized and transported through the ceramic membrane. Protons, having lower mass, migrate faster than deuterons, allowing partial isotope separation.^[^ ^152^ ^]^ Devices based on cerates leverage the isotope effect in ionic conductivity to distinguish between isotopes. The effectiveness improves under carefully controlled temperature, pressure, and gas composition.^[^ ^153^ ^]^ The difference in partial pressure between the retentate and permeate sides of the membrane drives the selective diffusion of hydrogen isotopes.^[^ ^154^ ^]^ The proton‐conducting perovskite membrane selectively allows H⁺ transport while blocking other species, enabling effective isotope separation and ultra‐pure hydrogen production. The resultant hydrogen can achieve remarkable purity levels (99.999999999%).^[^ ^155^ ^]^ Here, **Figure** **4** illustrates the D–T (Deuterium–Tritium) purification system using proton‐conducting oxide materials as isotope separators.^[^ ^143^ ^]^ A DC source drives the electrochemical separation process, where a gas mixture containing DTO (deuterium oxide), CT₄ (tritiated methane), CD₄ (deuterated methane), and He enters the system. Inside the proton‐conducting membrane, protons (T⁺ and D⁺) selectively migrate toward the cathode side due to the applied potential, based on their mass‐dependent mobility. On the cathode side, DT (deuterium‐tritium gas), D₂, and T₂ are formed and collected. Electrons flow through the external circuit while protons are transported through the membrane, achieving isotope separation. This mechanism efficiently separates hydrogen isotopes for fusion fuel and other applications.

SrCeO₃ is preferred over BaCeO₃ for membrane materials due to its stability. Though SrCeO₂‐based membranes have lower hydrogen permeability, it can be enhanced through high‐temperature sintering (≈1400 °C).^[^ ^156^ ^]^ The low electronic conductivity of proton conductors can be offset by incorporating metallic or electronically conductive oxides.^[^ ^157^ ^]^ These membranes maintain chemical stability under hydrogen isotope exposure.^[^ ^158^ ^]^ Cermet membranes that combine ceramics with metals like Ni offer both proton conductivity and fast electron conduction, enhancing membrane performance.^[^ ^78^ ^]^ Cerate‐based proton conductors, such as BaCe₀.₉Yb₀.₁O₂.₉₅ and SrCe₀.₉₅Yb₀.₀₅O₂.₉₇₅, are promising for hydrogen isotope separation due to their high proton conductivity and stability in water and CO₂ atmospheres.^[^ ^159^, ^160^ ^]^ A 2025 study demonstrated the use of cerate materials in a proton‐conducting fuel cell (PCFC) to achieve a separation factor of ≈3.8 for hydrogen and deuterium at 600 °C and 0.7 V, indicating strong potential for nuclear energy applications.^[^ ^126^ ^]^

### 3.3 Mechanism of Tritium Recovery Systems

**Figure 5:**
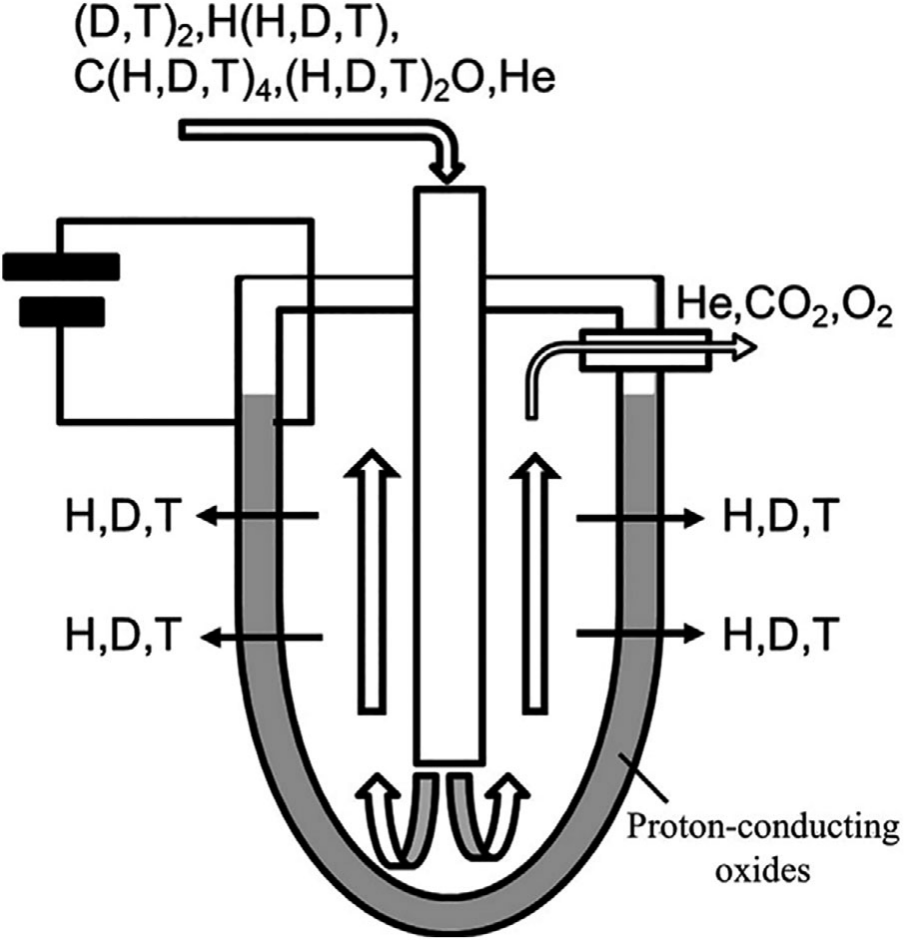
Schematic representation of a tritium recovery system employing proton‐conductive oxides. Reproduced with permission from ref. [91].

The research on fusion reactors that use nuclear fusion of heavy hydrogen with tritium is ongoing to create a clean energy source in the future. In a fusion reaction, the exhaust gas processing system must attain a high tritium decontamination factor, for example, using hydrogen recovery devices. Regarding the safety and security of a nuclear fusion reactor, a hydrogen isotope, tritium, loss from the exhaust gas processing system is a significant concern.^[^ ^91^ ^]^ Therefore, Perovskite PCOs are a promising candidate material in nuclear fusion reactors’ different electrochemical devices like the hydrogen sensor, tritium recovery system, hydrogen isotope separation, and hydrogen pump due to their excellent thermal and chemical stability and high H^+^ conductivity at high temperatures.^[^ ^91^, ^143^ ^]^ A conceptual diagram of the tritium recovery system is presented in **Figure** **5**.^[^ ^91^ ^]^ Figure 5 shows a tritium recovery system using proton‐conductive oxides.^[^ ^91^ ^]^ A gas mixture containing hydrogen isotopes (H, D, T) and other species is introduced into the chamber. Under applied voltage, protons (including tritons) migrate through the proton‐conducting oxide membrane. The recovered tritium exits as purified gas, while other gases like He, CO₂, and O₂ are removed.

The tritium recovery system that uses the same principles as a hydrogen pump can separate the hydrogen isotope from the plasma exhaust gas, including contaminants such as DTO, He, CD_4_, and CT_4_.^[^ ^91^ ^]^ When DC power is delivered between the electrodes of this tritium recovery system (Figure 5), purified deuterium (D), tritium (T), DT, D_2_, and T_2_ are produced.^[^ ^161^ ^]^ The production of these elements can be recycled as fuel in the fuel circulation system of a nuclear fusion reactor.^[^ ^71^ ^]^ Cerate‐based perovskite proton conductors are widely used in tritium purification and recycling due to their excellent mechanical and electronic properties. Compared to the other cerate‐based proton conductor, doped strontium cerates with ytterbium like SrCe_0.95_Yb_0.05_O_3‐α_ are used as an electrochemical cell for the tritium recovery system application due to their high tritium conductivity with good chemical stability at high temperature.^[^ ^162^ ^]^ A high temperature is expected for tritium extraction in the tritium recovery system of a fusion reactor.

Cerate‐based electrochemical hydrogen devices have demonstrated promising results in tritium recovery systems. Perovskite‐type ceramics, such as CaZr₀.₉In₀.₁O₃_−α_ and SrZr₀.₉Yb₀.₋O₃_−α_, is suitable for tritium extraction in fusion reactors because they have strong proton conductivity and good chemical stability at high temperatures.^[^ ^56^ ^]^ In CaZr₀.₉In₀.₁O₃_−α_, one‐end closed tubes have been used in experiments to recover hydrogen with efficiency above 99%. Almost 100% current efficiency was also demonstrated in these tests, which were carried out at 1023 K and 1.15 V, demonstrating the material's ability to separate hydrogen isotopes from gas mixtures that are rich in helium.^[^ ^128^ ^]^ The choice of electrode material greatly affects performance. For example, plated platinum electrodes work best with gas mixtures containing hydrogen, water vapor, and methane, while porous pasted platinum electrodes are more effective for water vapor electrolysis, as shown in studies under different conditions.^[^ ^163^ ^]^ The design of electrochemical cells is important for improving tritium recovery. Larger electrode areas allow for higher current densities, which lead to better extraction efficiency.^[^ ^56^, ^164^ ^]^

### 3.4 Mechanism of Hydrogen Sensors

**Figure 6:**
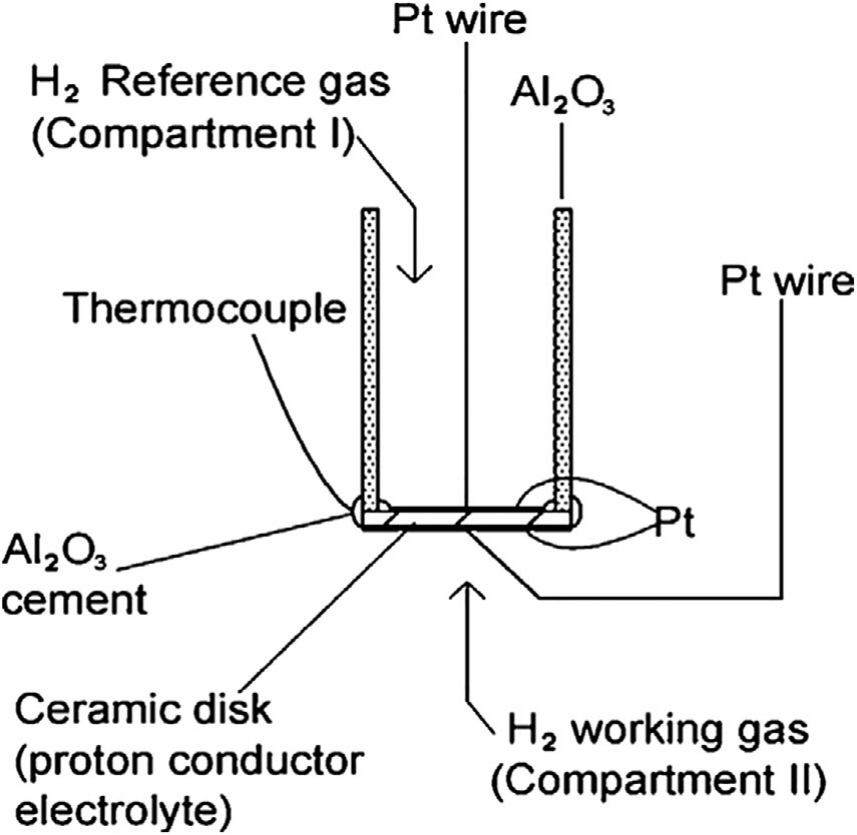
A pictorial presentation of the hydrogen sensor. Reproduced with permission from ref. [165].

Hydrogen has immense combustion heat (142 KJ g^−1^ H_2_^−1^), minimum flammable energy (0.017 mJ), a broad range of flames (4–75%), high ignition temperature (560 °C),  and maximum flaming velocity. Humans cannot identify hydrogen gas since it has no taste, color, or odor. Due to its low ignition energy and broad flammable range, hydrogen is easily flammable and explosive. As a result, developing a reliable and accurate hydrogen sensor is necessary to prevent damage and explosions in several applications. A hydrogen sensor is a device that can detect hydrogen at concentrations close to 100% and at parts per million (ppm) levels that are close to the lower explosive limit (4% H_2_ in the atmosphere). Hydrogen sensors are available in electrochemical devices, semiconductors, thermoelectrics, etc. A schematic diagram of the hydrogen sensor is demonstrated in **Figure** **6**.^[^ ^165^ ^]^ This figure illustrates a hydrogen sensor setup based on a **proton‐conducting ceramic electrolyte disk**. It features two compartments: **Compartment I** with H₂ reference gas and **Compartment II** with the H₂ working gas. **Pt wires and electrodes** are used to establish electrical contact and facilitate proton conduction across the ceramic disk. A **thermocouple** is included to monitor the operating temperature, which is crucial for proton conductivity. **Al₂O₃ cement and tubes** are used to support and seal the structure, ensuring gas‐tight operation and stability. The development of highly proton‐conductive ceramics is essential for efficient hydrogen sensors. Cerate‐based oxides like BaCe₀.₇Zr₀.₁Y₀.₂O₃−δ, BaCe₀.₈Gd₀.₂O₃ (BCG), and BaZr₀.₄Ce₀.₄In₀.₂O₃ (BZCI) exhibit high proton conductivity and thermal stability, making them suitable electrolytes for potentiometric and amperometric hydrogen sensors.^[^ ^166^, ^167^, ^168^ ^]^ Materials such as SrCeO₃ and CaZrO₃ also perform well at high temperatures in hydrogen‐rich environments.^[^ ^169^ ^]^ These oxides quickly respond to H₂ by proton generation and conduction, enabling real‐time, sensitive detection of hydrogen. Their compactness and affordability allow usage in portable sensors for applications like refueling stations, hydrogen production, and automotive monitoring.

Hydrogen sensors utilizing cerate‐based materials have demonstrated significant advancements in electrochemical hydrogen detection. For instance, a study used polyaniline/cerium oxide (PANI/CeO₂) nanocomposites to develop an effective electrochemical sensor. These nanocomposites demonstrated outstanding electrocatalytic oxidation properties toward hydrogen peroxide, indicating that these materials could be used in hydrogen sensing applications.^[^ ^170^ ^]^ Another study examined glassy carbon electrodes modified with nano‐structured CeO₂. The increased surface area and oxygen vacancies in the nanostructured material have been demonstrated to be responsible for the improved electrochemical sensing behavior for hydrogen peroxide reduction.^[^ ^171^ ^]^ Furthermore, a cerium oxide nanocube‐based nonenzymatic electrochemical sensor demonstrated rapid and precise detection of hydrogen peroxide residues in food samples, emphasizing the adaptability of cerate materials in sensor applications.^[^ ^172^ ^]^

## 4 Literature Review and Performance Analysis of Cerate Material‐Based Electrochemical Hydrogen Devices

The following sections represent the recent developments in the synthesis process, performance, and improved properties of cerate‐based materials as electrolytes in electrochemical hydrogen devices named hydrogen sensors, tritium recovery systems, hydrogen isotope separation systems, and hydrogen pumps.

### 4.1 Performance Analysis of Cerate‐Based Hydrogen Pumps

**Figure 7:**
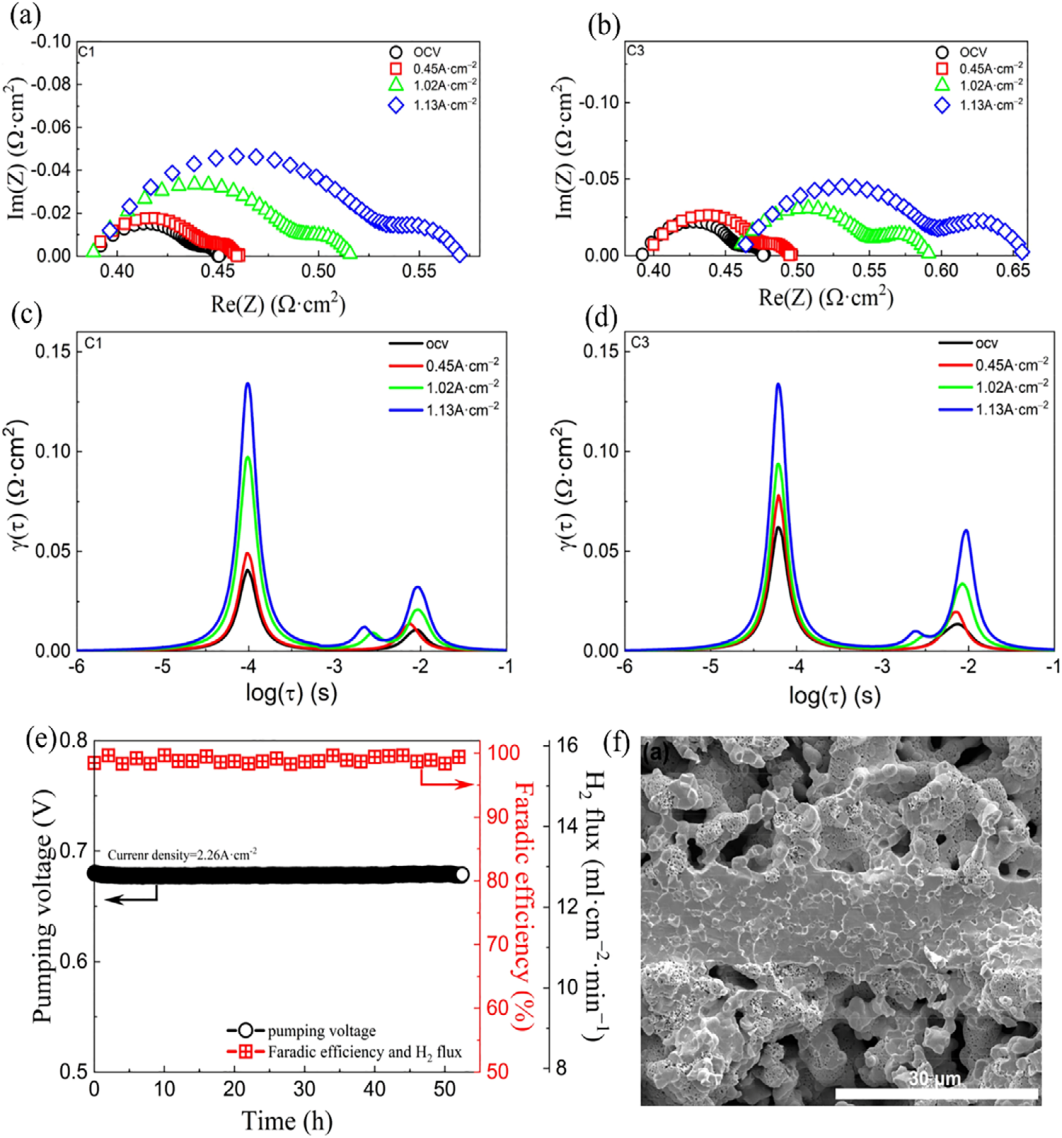
Impedance data were measured for the ceramic hydrogen pump operating under the gradient of 10% H2/N2|| 100% H2 within both sweep and feed gases and dry feed side configurations. a–d) Nyquist plots (a, c) and DRT profiles (b, d) are shown. e) The measurements of the ceramic hydrogen pump's short‐term stability to extract pure H2 from 50% H2/N2 at 500 °C, and f) a cross‐section of SEM for the ceramic hydrogen pump. Reproduced with permission from ref. [40]

Doped BaCeO_3_ materials have been investigated as solid electrolytes in hydrogen pump applications because of their strong proton conductivity and chemical stability in harsh environments. Patki et al. investigated a BaZr_0.8_Ce_0.1_Y_0.1_O_3‐δ_‐based membrane for galvanic hydrogen pumping in the methane dehydroaromatization reaction for in‐situ separation of product hydrogen from the system.^[^ ^82^ ^]^ They used a BaZr_0.8_Ce_0.1_Y_0.1_O_3‐δ_ ‐cermet as a cathode, and electroless plated Cu onto perovskite materials to serve as an anode electrode on one side. The performance of such a system under a gas mixture containing methane, ethane, CO, and H_2_ demonstrates that the proposed system is capable of pumping H_2_ at 40 mA cm^2^ through Cu─Cu electrodes using 268 mW/(NmL H_2_ min^−1^); and the faradaic efficiency decreases with increasing cerium content in the solid membrane structure, possibly due to cerium migration from cermet structure. Recently, Mushtaq et al. developed a reliable and efficient hydrogen pump with BaZr_0.7_Ce_0.2_Y_0.1_O_3‐δ_ (BZCY271) electrolyte by using a vacuum‐assisted dip coating approach on a porous Ni‐BZCY support.^[^ ^147^ ^]^ Another hydrogen pump demonstrated by Tong et al. using porous BaZr_0.1_Ce_0.7_Y_0.2_O_3‐δ_cermet instead of a metal electrode significantly enhanced the H_2_ flux (15.6 mL cm^−2^ min^−1^) at 500 ⁰C and made them suitable for low‐temperature (350–500 ⁰C) hydrogen pumping applications.^[^ ^140^ ^]^ **Figure** **7**a,c displays Nyquist plots and Figure 7b,d displays the distribution of Relaxation Time (DRT) of impedance data obtained under the gradient of 10% H_2_/N_2_||100% H_2_ for the ceramic hydrogen pump with wet in both sweep and feed gases, and dry in the feed side. The dynamics of hydrogen adsorption and desorption are better understood in the context of a hydrogen pump by DRT spectra. For understanding the surface reactions, charge transfer, and diffusion processes related to hydrogen pumping by examining the distribution of relaxation times.^[^ ^173^ ^]^ Impedance measurements here revealed that under these conditions, the resistance at the electrode/electrolyte interface increases significantly at high current densities. This suggests a decrease in the concentration of a key component (OH: O) at this interface. Additionally, for cells with a limited hydrogen supply, the overall resistance jumps at a critical current density, potentially due to a switch in the reaction mechanism at the anode. These findings suggest that a sufficient supply of hydrogen is crucial for maintaining efficient fuel cell operation. Figure 7e shows the measurement of the short‐term stability of the ceramic hydrogen pump as it extracts pure H_2_ at 500 °C from a mixture of 50% H_2_/N_2_. A cross‐section of the SEM micrograph for the ceramic hydrogen pump is shown in Figure 7d.^[^ ^140^ ^]^

Complex oxide conducting materials having dominant ion transportation characteristics compared to electronic transportation have seen prospective applications as electrolyte materials while designing an EHP.^[^ ^174^ ^]^ The infiltration technique has opted to design an efficient EHP possessing the general structure Ba(Zr_0⋅85_Y_0.15_)O_3‐δ_‐(La_0⋅7_Sr_0⋅3_)V_0.9_O_3‐δ_@CeO_2_‐Pd**|**Ba(Zr_0.30_Ce_0.54_Y_0.15_Cu_0.01_)O_3‐δ_ **|**Ba(Zr_0⋅85_Y_0.15_)O_3‐δ_‐(La_0⋅7_Sr_0⋅3_)V_0.9_O_3‐δ_@CeO_2_‐Pd.^[^ ^145^ ^]^ Here, the Ba (Zr_0.30_Ce_0.54_Y_0.15_Cu_0.01_)O_3‐δ_ ceramic acts as the electrolyte material, whereas the Ba(Zr_0⋅85_Y_0.15_)O_3‐δ_ and (La_0⋅7_Sr_0⋅3_)V_0.9_O_3‐δ_ ceramics perform as the protonic and electron conducting composite electrodes, respectively, with CeO_2_ and Pd working as the catalysts. The hydrogen pump exhibited a significant 2 Acm^−2^ current density, having a meager 1.1 V overpotential owing to the presence of the composite ceramic electrodes. The electron‐conducting anode (La_0⋅7_Sr_0⋅3_)V_0.9_O_3‐δ_ opted in this hydrogen pump is suitable for operation under oxygen partial pressure <10^−17^ and has been utilized in galvanic hydrogen pumps and PCFC as well.^[^ ^175^ ^]^ Another PCO, SrCe_0.95_Yb_0.05_O_3‐α_ has been utilized for hydrogen extraction through electrochemical pumps.^[^ ^176^ ^]^ A direct current having approximately unity efficiency in this system has been observed to pump pure hydrogen entirely from the anode to the cathode side from a synthesis gas mixture of H_2_ and CO at a high temperature of 800 °C. The water vapor electrolysis increased the current density and decreased the ionic transportation number due to partial n‐type conductivity.^[^ ^145^ ^]^ By comparing the performances of SrCe_0.95_Yb_0.05_O_3‐α_ with SrZr_0.95_Yb_0.05_O_3‐α_, SrCe_0.95_Yb_0.05_O_3‐α_ demonstrated superior electrochemical performances in terms of proton conduction in hydrogen pumps.^[^ ^83^ ^]^ Matsumoto et al. also observed that proton conductivity through SrCe_0.95_Yb_0.05_O_3‐α_ electrolyte was enhanced due to humidity in the feed gas at the cathode within a temperature range of 700–900 ⁰C.^[^ ^88^ ^]^

Kawamura et al. proposed a novel technique to enhance the transportation of hydrogen by using a triple‐phase boundary (region among gas phase, electrode, and electrolyte) system between electrolyte and electrodes. The sputtering technique was implemented to fabricate Pt and Pd electrode layers and subsequent attachment to the disk‐shaped ceramics (SrCe_0.95_Yb_0.05_O_3‐α_) as electrolytes. In comparison between the two electrode materials, due to the high hydrogen permeability, the current density in the nonporous Pd electrode is much higher (4–5 times) than in the traditional Pt electrode. In addition, using a sputtering electrode compared to a paste electrode was reported to be beneficial for enhancing the current density, resulting in hydrogen transportation capability. A comparison of an electrode, gas flow rate, applied voltage, and temperature between Pt and porous Pt type electrode with SrCe_0.95_Yb_0.05_O_3‐α_ electrolyte are presented in **Table** **3**.^[^ ^162^, ^177^ ^]^ It is observed that H_2_, H_2_/He, and helium flow rates are 0.4 L min^−1^ with 0 to 2.0 V at 873 K when the Pt electrode is used, whereas H_2_–D_2_/He, HT–H_2_/He flow rates are 0.2 and 0.4 0.4 L min^−1^, with 0 to 1000 mV at 873 K when porous Pt electrode is used in the hydrogen pump (Table 3).^[^ ^177^ ^]^ Matsumoto et al. have investigated the electrochemical hydrogen pumping efficiency of pure hydrogen gas for SrCe_0.95_Yb_0.05_O_3‐α_ as a proton‐conducting solid electrolyte at high temperature (900 °C).^[^ ^178^ ^]^ The current efficiency decreased at high current density due to the critical current density reducing efficiency, and decreased with the rising amount of water vapor in the anode hydrogen gas. The hydrogen inadequacy and the increase of oxygen partial pressure at the anode in sending high current typically account for the partial hole conduction in the electrolyte and the reduction of the current efficiency of the hydrogen pump.^[^ ^179^ ^]^

**Table 3:** Cerate‐based hydrogen pump systems with electrode type, sample gas, flow rate, applied voltage, and temperature.

Electrolyte materials	Electrode type	Sample gas	Flow rate [L min^−1^]	Applied voltage [V]	Temperature [K]	Stability under CO₂/H₂O	Refs.
SrCe_0.95_Yb_0.05_O_3‐α_	Pt	H_2_, H_2_/He, & helium	0.4	0 to 2.0	873	N/A	[162]
SrCe_0.95_Yb_0.05_O_3‐α_	Porous Pt	H_2_–D_2_/He	0.2, 0.4	0 and 0.1	873	N/A	[177]
		HT–H_2_/He	0.2 (0.12)				
SrCe_0.95_Yb_0.05_O_3‐α_	Porous Pt	Ar and H_2_	0.03	0.25–1.0	1173.15	N/A	[142]
SrCe_0.95_Yb_0.05_O_3‐α_	Porous Pt	Ar and H_2_	0.06–0.1	1.5–2.0	923.15–1073.15	N/A	[179]

### 4.2 Performance Analysis of Cerate‐Based Hydrogen Isotope Separation Systems

**Figure 8:**
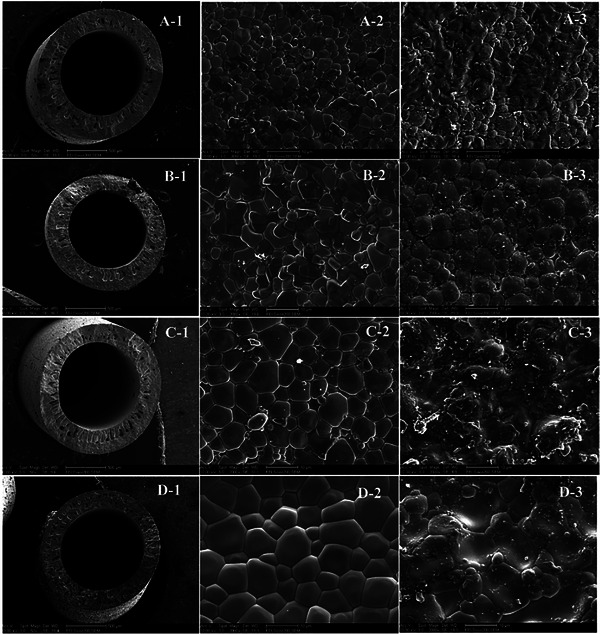
SEM images of BaCe0.85Tb0.05Co0.1O3‐δ hollow fiber membranes sintered at temperatures of 1200, 1300, 1400, and 1500 °C (1: cross‐section; 2:inner surface; 3: outer surface). Reproduced with permission from ref. [181].

The effective way of promoting hydrogen permeation flux is by reducing the membrane thickness and the sintering process of the membrane. *Xiaoyao Tan* et al. have fabricated a gas‐tight hollow fiber membrane consisting of BaCe_0.95_Tb_0.05_O_3‐α_ (BCTb) perovskite oxides by the combined use of phase inversion and steering technique at elevated temperature.^[^ ^180^ ^]^ Based on the final crystal morphology and porosity investigated by SEM images, the sintering temperature range of 1300–1500 °C is ideal for fabricating a BCTb‐based gas‐tight membrane. The hydrogen permeability increased steadily with the measurement temperature (700–1000 °C) and sweep gas (N_2_) flow rates. However, Transition metal cations such as cobalt, having variable valance properties, have been introduced into the ceramic membrane to achieve higher electronic conductivity and improve the hydrogen permeation flux.^[^ ^181^ ^]^ Through a combination of phase inversion and sintering methods, cobalt‐doped BaCe_0.85_Tb_0.05_Co_0.1_O_3‐δ_ hollow membranes were synthesized. Since the sintering properties of perovskite ceramic and transition metal phases differ significantly, synthesizing hollow tubular fibers through this technique is complex and requires additional attention.^[^ ^182^ ^]^ The cobalt‐doped BaCe_0.85_Tb_0.05_Co_0.1_O_3‐δ_ exhibited higher electrical conductivity than the BaCe_0.95_Tb_0.05_O_3‐δ_ oxide, which may be attributed to the rise in oxygen ionic as well as electronic conductivity. The maximal hydrogen permeation flux through BaCe_0.85_Tb_0.05_Co_0.1_O_3‐δ_ hollow fiber membranes can reach 0.385 mL cm^−2^ min^−1^ at 1000 °C when the flow rates the 50% H_2_‐He/N_2_ are 60 and 100 mL min^−1^, respectively (**Table** **4**).^[^ ^181^ ^]^ The development of the BaCe_0.95_Tb_0.05_O_3‐δ_ hollow fibers' microstructure with the sintering temperature is shown in **Figure** **8**.^[^ ^181^ ^]^ It is observed that the general structure of ceramic hollow fibers does not change significantly at a high sintering temperature (Figure 8). The inner surface micrographs, however, clearly show that the sintering temperature considerably impacted the grain growth in the membrane.

**Table 4:** Different types of cerate‐based hydrogen isotope separation systems with different synthesis processes, membrane structure, temperature, atmosphere, and maximum hydrogen flux.

Electrolyte materials	Synthesis method	Membrane structure	Temperature [°C]	Operating atmosphere	Stability under CO₂/H₂O	Maximum hydrogen flux [mL cm^−2^ min^−1^]	Refs.
BaCe_0.85_Tb_0.05_Co_0.1_O_3‐δ_	Combined phase inversion and sintering technique	N/A	1000	50% H_2_‐He/N_2_	N/A	0.385	[181]
Ni‐Ba(Zr_0.1_Ce_0.7_Y_0.2_)O_3‐δ_	N/A	N/A	900	80% H_2_‐N_2_/Argon		0.322	
BaCe_0.95_Nd_0.05_O_3‐δ_	N/A	0.7 mm thick	925	80% H_2_/He		0.017	
BaCe_0.95_Tb_0.05_O_3‐δ_	N/A	N/A	1000	50% H_2_‐He/N_2_		0.57	
Ni‐BCTb	Isostatic pressing	N/A	850	50% H_2_‐N_2_/He		0.914	
BaCe_0.9_Mn_0.1_O_3‐δ_	N/A	1.06 mm thick	900	80% H_2_/He		0.015	
BaCe_0.8_Y_0.2_O_3‐ä_	Phase inversion and sintering method	Gas tight hollow fiber	1050	50% H_2_‐He/N_2_	N/A	0.38	[183]
BaCe_0.8_Y_0.2_O_3‐ä_	Statically pressing and sintering	Disk shaped	950	50% H_2_‐He/N_2_		<0.01	
Ni‐BaCe_0.8_Y_0.2_O_3‐δ_	N/A	80‐mm‐thick	N/A	N/A	Stable	0.25	[184]
Ni‐BaZr_0.1_Ce_0.7_Y_0.2_O_3‐δ_	Co‐pressing	30 mm asymmetric	900	N/A		0.33	
NiO‐BaZr_0.1_Ce_0.7_Y_0.1_Yb_0.1_O_3‐δ_	Particle suspension coating	44 µm	900	Pure H_2_/N_2_		1.12	
	Particle suspension coating	N/A	700	Pure H_2_/N_2_		0.49	
BaCe_0.95_Tb_0.05_O_3−α_	Phase inversion and sintering technique	Gas tight hollow fiber	1000	H_2_‐He/N_2_	N/A	0.57	[78]
	Phase inversion and sintering technique	Gas tight hollow fiber	700	H_2_‐He/N_2_		0.3	
SrCe_0.95_Y_0.05_O3‐δ–ZnO [SCY‐10% ZnO]	N/A	1.09 mm‐thick dual‐phase membranes	900	21% H_2_‐He/N2	N/A	0.039	[185]
Ni–BaZ_r0.1_Ce_0.7_Y_0.2_O_3−α_	Solid‐state reaction, cold isostatically pressing & sintering	Pellets with 15 mm diameter & 0.5 mm thickness	N/A	Pure H_2_‐pure N_2_‐ H_2_S/Argon	N/A	N/A	[180]

**Figure 9:**
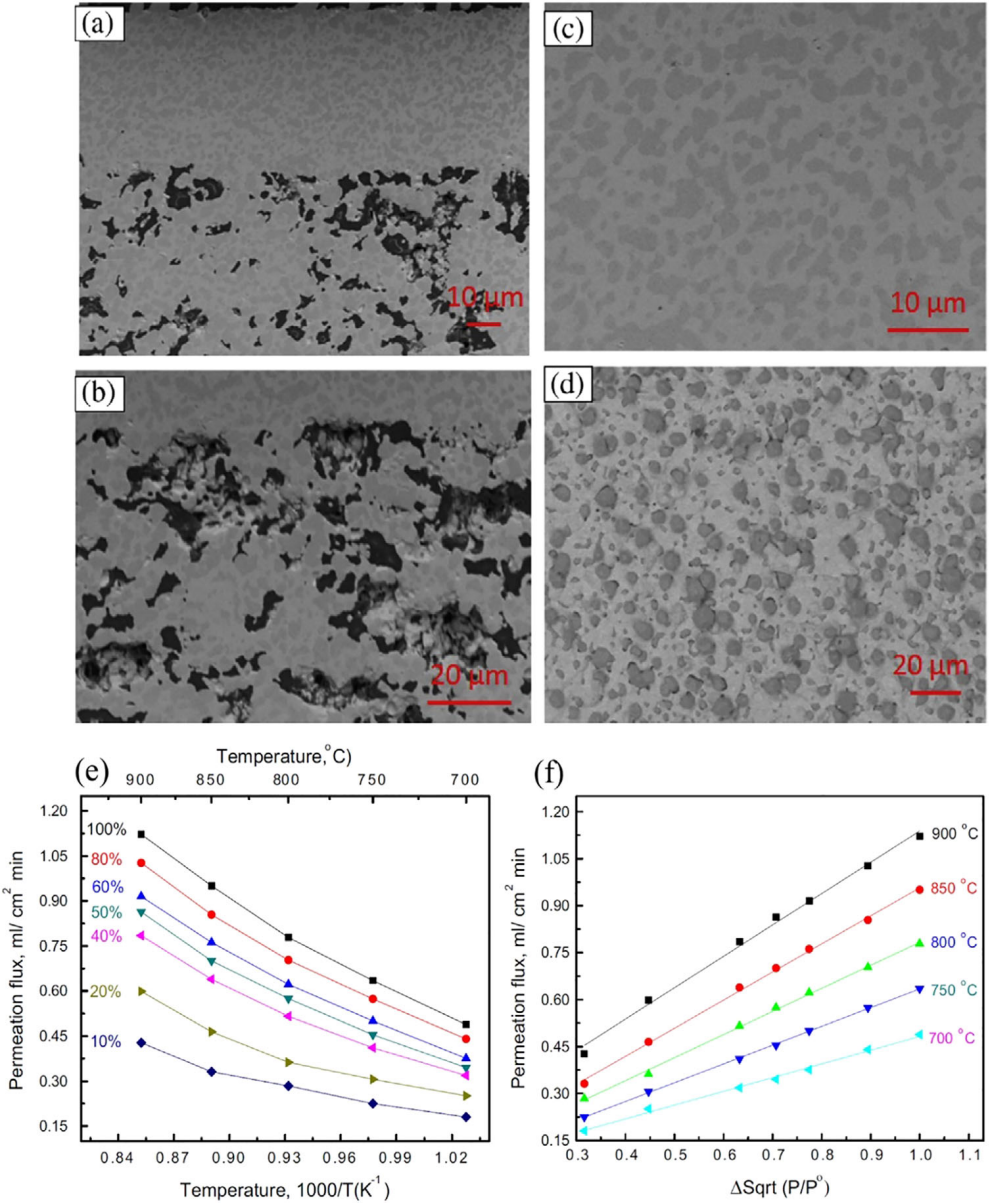
a) An SEM image of the cross‐sectional view of a dense Ni‐BZCYYb membrane on a porous Ni‐BZCYYb substrate; b) a high magnification SEM image of the interface between the dense membrane and porous substrate; c) a high magnification SEM image of the dense Ni‐BZCYYb membrane; and d) backscattered electron images of the surface. e) H_2_ permeation fluxes of the Ni‐BZCYYb membrane as constructed at various hydrogen partial pressures in the feed gas and temperatures during operation. f) The impact of H2 partial pressure on the thin membrane's hydrogen flux. Reproduced with permission from ref. [78].

The Ni‐BZCY (Ni–BaZr_0.1_Ce_0.7_Y_0.2_O_3−_ *_δ_*) exhibited excellent stability and performance in the presence of H_2_O and CO_2_ at high temperatures (900 °C), whereas unstable in the case of acidic H_2_S. Fank et al. reported the chemical stability of Ni‐BZCY and the hydrogen permeation flux in the presence of H_2_S concentrations of 30–300 ppm at 900 °C to overcome the instability mentioned above.^[^ ^184^ ^]^ The steady hydrogen permeation flux (HPF) of Ni‐BZCY cermet (ceramic‐metal composite) was found in the presence of 30 ppm H_2_S; however, it started to decrease in the presence of 60 ppm H_2_S. Liu et al. have fabricated a thin Ni‐BZCCYb (Ni incorporated on BaZr_0.1_Ce_0.7_Y_0.1_Yb_0.1_O_3‐δ_) membrane on porous Ni‐BZCYYb substrate support via a co‐firing process.^[^ ^78^ ^]^ The hydrogen separation was at temperature ranges from 700 to 900 °C, and hydrogen permeation fluxes under different flux conditions of 1.12 and 0.49 mL min^−1^ cm^−2^ at 900 and 700 °C, respectively. **Figure** **9**a,b shows a cross‐sectional view of a dense Ni‐BZCYYb membrane on a porous Ni‐BZCYYb substrate.^[^ ^78^ ^]^ Figure 9c presents dense Ni‐BZCYYb membranes, and Figure 9d shows a backscattered electron image of a dense Ni‐BZCYYb membrane. The permeation flux with temperature (Figure 9e) and the permeation flux with H_2_ partial pressure (Figure 9f) are presented. It is noticed in Figure 9a–d that the metal phase in the BZCYYb matrix is randomly dispersed, and the membrane is dense. The nickel phase in this cermet membrane significantly increases the cermet's H_2_ permeability through enhancement of the cermet's electronic conductivity, surface exchange kinetics, and mechanical stability. Figure 9e presents that the H_2_ permeation rate is higher than the cermets' previously published H_2_ permeation rate.^[^ ^78^ ^]^

Tan et al. synthesized BaCe_0.8_Y_0.2_O_3‐δ_ hollow membranes with the addition of 1 wt% sintering aid (Co_2_O_3_) by following phase inversion and sintering techniques.^[^ ^183^ ^]^ BaCe_0.8_Y_0.2_O_3−δ_ is a composite material belonging to the rare‐earth perovskite family. It's made by partially substituting cerium (Ce) with yttrium (Y) in barium cerate (BaCeO_3_). The ‐δ notation signifies oxygen vacancies, which can be adjusted to influence their properties. The hollow membranes are specifically designed with a hollow fiber structure. Imagine a long, thin tube with a hollow center. This design increases the surface area for gas permeation, making them efficient for gas separation processes. The best mechanical strength of the BaCe_0.8_Y_0.2_O_3−δ_ hollow fiber membrane was found at the ideal sintering temperature of 1400 °C. In addition, the maximum H_2_ permeation flow through the BaCe_0.8_Y_0.2_O_3−δ_ hollow fibers reached up to 0.38 mL cm^−2^ min^−1^ at 1050 °C (Table 4), which is noticeably in contrast to the lower value of less than 0.01 mL cm^−2^ min^−1^ of BaCe_0.8_Y_0.2_O_3−δ_ disk‐shaped membrane.^[^ ^183^ ^]^ To meet the requirement of high electronic conductivity, Wang et al. have proposed metal oxide (Zinc Oxide‐ ZnO) mixed with the protonic conductor phase of SrCe_0.95_Y_0.05_O_3_ to form a dual phase membrane for permeation of hydrogen in high‐temperature.^[^ ^185^ ^]^ The following reason is mentioned for choosing ZnO: i) Zinc oxide is expected to lower the sintering temperature rather than the expense of proton conductivity, and ii) As a dopant, to improve electronic conductivity of oxygen ionic conductor, the ZnO is used over a wide range of temperature and partial pressures of oxygen,^[^ ^186^ ^]^ iii) The thermal expansion coefficient of ZnO is almost identical in comparison with SrCeO_3_ that reduced the formation of crack, and it is low cost, nontoxic and stable material in chemically with availability in abundance at nature, and finally; (iv) ZnO increases the sintering ability of SCY perovskites which reduced the sintering temperature. A variation of the ZnO amount in the composite was carried out. A composite having 90% SCY‐10% ZnO was found to be optimum, as it shows good stability after 48 h at 900 °C, and the peak flux of hydrogen permeation is found to be 0.039 mL (STP) cm^−2^ min^−1^.

Kawamura et al. have studied the EHP where SrCe_0_._95_Yb_0.05_O_3‐α_ proton conductor is used as a membrane within a blanket breeder system to extract the isotopes of hydrogen, including tritium, from water decomposition.^[^ ^177^ ^]^ The EHP has the advantage of selectively extracting hydrogen isotopes from sweep gas due to its electrostatic potential distinction driving force.^[^ ^73^ ^]^ It is reported that a balanced H_2_‐D_2_/He or HT‐H_2_/He sample gas mixture was supplied to EHP at an operating condition of 600 °C with a varied applied voltage between 0 and 1000 mV. Mukundan and his coworker investigated BaCe_0.9_Yb_0.1_O_2.95_, SrZr_0.9_Yb_0.1_O_2.95_, and SrCe_0.95_Yb_0.05_O_2.975_ PCOs by using ac impedance analysis.^[^ ^160^, ^187^ ^]^ The BaCe_0.9_Yb_0.1_O_2.95_ proton conductor exhibited the highest proton occupancy compared to the SrZr_0.9_Yb_0.1_O_2.95_ and SrCe_0.95_Yb_0.05_O_2.975_ proton conductors. The SrCe_0.95_Yb_0.05_O_2.975_ proton conductor exhibited the lowest protonic conductivity and thereby 2.5% vacancies for oxygen, where protons may be incorporated in a wet state. The activation energy was observed to demonstrate a large scatter, which may be attributed to the varying proton concentration with varying temperature and grain boundary conductivity. The high tritium conductivities observed here for BaCe_0.9_Yb_0.1_O_2.95_ and SrZr_0.9_Yb_0.1_O_2.95_ proton conductor membranes make them especially suitable for use in tritium separation. Table 4 represents the different types of cerate‐based electrolytes with synthesis process, membrane structure, temperature, operating atmosphere, and maximum hydrogen flux.^[^ ^78^, ^180^, ^181^, ^183^, ^184^, ^185^ ^]^

### 4.3 Performance Analysis of Cerate‐Based Tritium Recovery Systems

**Figure 10:**
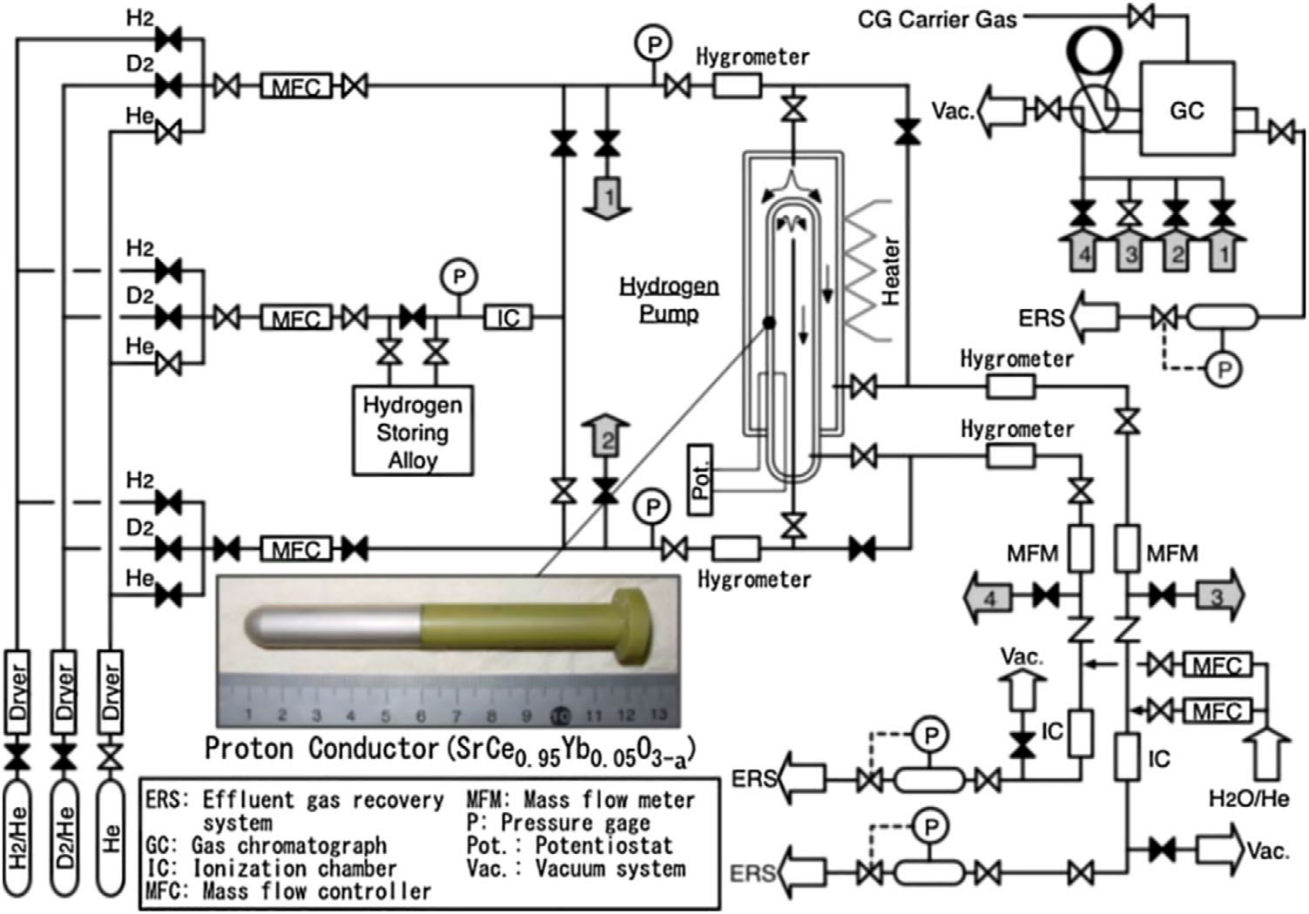
An illustration of the experimental setup. Reproduced with permission from ref. [177].

**Figure 11:**
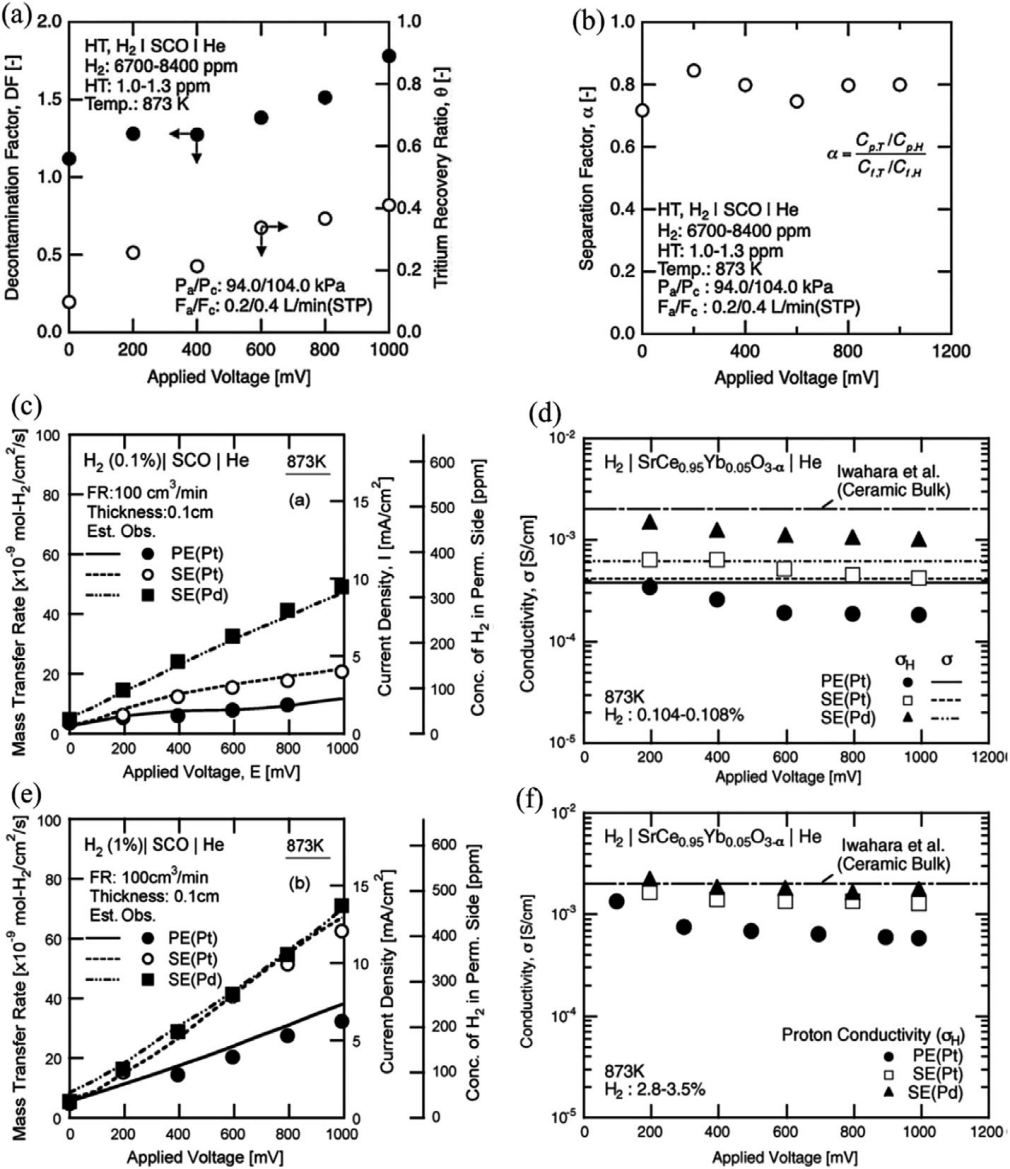
a) Tritium recovery ratio and decontamination factor for different voltage applications. And b) Tritium enrichment for a range of applied voltages. Reproduced with permission from ref. [177] c,e) comparing the current‐voltage characteristics of several electrodes (d), f) the comparison of the conductivities among various electrodes. Reproduced with permission from ref. [74].

Membrane permeation, such as Pd‐Ag, has been utilized in exhaust treatment and processing while designing tritium recovery systems.^[^ ^188^ ^]^ However, using Pd‐Ag membrane permeation faces difficulty in the system involving low tritium partial pressure, incorporating water vapor processing.^[^ ^189^ ^]^ Kawamura et al. have utilized a hydrogen pump with proton‐conducting SrCe_0.95_Yb_0.05_O_3‐α_ ceramic membrane to extract the hydrogen isotopes from He sweep gas only for an H_2_–H_2_O gas mixture in a blanket tritium recovery system.^[^ ^162^ ^]^ Sufficient electrical potential difference enables selective extraction of hydrogen isotopes from the blanket sweep gas through low partial to high partial pressure, facilitating tritium recovery from tritiated hydrocarbons and water molecules. Such a compact system is effective in low tritium partial pressure environments where He sweeps gas has a relatively large flow rate. Furthermore, Kawamura et al. studied the transport properties of the hydrogen isotopes for multicomponent sweep gas in a later work.^[^ ^177^ ^]^ It is observed that the hydrogen isotopes are dissociated and then absorbed on the electrode, followed by diffusion through the lattice structure. Such a system demonstrated ≈40% bred tritium extraction ability through the SrCe_0.95_Yb_0.05_O_3‐α_ ceramic proton conducting permeator upon application of an electric potential in the range of 800 mV. **Figure** **10** shows a diagram of experimental equipment.^[^ ^177^ ^]^ A voltage (from 0 to 100 mV) was applied between the electrodes to transport hydrogen isotopes from the interior to the exterior of the SrCe_0.95_Yb_0.05_O_3‐α_ tube. In the HT‐H_2_ system, the sample gas was supplied by flowing He gas through an alloy bed that was used to contain a mixture of tritium and hydrogen. **Figure** **11**a,b represents the Tritium recovery ratio and decontamination factor for different voltage applications and Tritium enrichment for various applied voltages.^[^ ^177^ ^]^ The comparison between current‐voltage properties and conductivity of different electrodes is shown in Figure 11c–f.^[^ ^74^ ^]^

The use of S_r_Ce_0.95_Yb_0.05_O_3‐α_ as electrolyte for tritium pump/separation process in nuclear fusion technology due to excellent tritium recovery performance from low concentration tritium‐containing water vapor in blanket sweep gas.^[^ ^162^ ^]^ A comparative study based on both experimental and empirical investigation was carried out by Fukuda et al. to compare properties such as total impedance and diffusion impedances.^[^ ^190^ ^]^ In such an application, the transfer of both mass and charge is generally explained by the total impedance plot, termed the cole‐cole plot. However, Fukada et al. studied Sr‐Ce‐Yb oxide‐based CH_4_+H_2_O|Ni|SrCe_0.95_Yb_0.05_O_3‐α_|NiO|O_2_+H_2_O system, having a porous Nickel electrode as an internal reformer without an additional methane reformer.^[^ ^191^ ^]^ The principal process on the electrode surface was stated to be the steam reforming reaction, and no traces of carbon deposits were found on the Ni electrodes. Though only H_2_ diffused to the cathode side through the SrCe_0.95_Yb_0.05_O_3‐α_ ceramic, specific volumes of CH_4_ underwent direct decomposition at the ceramic‐electrode interface following diffusion through the porous Ni electrode. Compared to the H_2_+H_2_O supply, protonic conductivity for the CH_4_+H_2_O supply was ten‐fold smaller. Here, the low electric conductivity of the ceramic impeded the conclusive identification of carbon localized on the ceramic surface. The relatively easy recovery of oxidized tritium as tritium water vapor on the anode side and future H_2_ or CH_4_ sensor applications make this system particularly advantageous.^[^ ^192^ ^]^

Kawamura et al. (2002)^[^ ^73^ ^]^ have investigated transfer characteristics of ionic deuterium versus hydrogen of the H_2_‐D_2_ mixture in a blanket tritium recovery (BTR) system consisting of SrCe_0.95_Yb_0.05_O_3−_ *_α_* membrane‐based hydrogen pump. Similar work was reported by Kakuta et al.^[^ ^193^ ^]^ on the use of a protonic conductor membrane (PCM) for the BTR system. In such a system, the electric potential difference is the driving force of hydrogen transportation through PCM. Therefore, EMF is measured from electrodes after attaching them to the PCM membrane's anode and cathode sides, with the addition of sensing and recording the different hydrogen partial pressures, applying the Nernst equation.^[^ ^193^, ^194^ ^]^ For the experiment, the SrCe_0.95_Yb_0.05_O_3‐α_ membrane is used as PCM, where a disk‐type membrane is utilized for impedance and a test tube‐type membrane for transportation properties measurement at 873 and 1073 K. Although the SrCe_0.95_Yb_0.05_O_3‐α_ membrane can be successfully used in such cases, an analytical model‐based study classifies the mass transportation phenomena through electrolyte membranes. Kawamura et al.^[^ ^195^ ^]^ have proposed the equation of the basic mass transfer rate of hydrogen by using the conductivity of apparent proton as a coefficient of mass transfer, where the estimation of the basic mass transfer of hydrogen was formulated with the help of apparent proton conductivities, with the implementation of the proposed equations and experimental data. The characteristics of ionic hydrogen regarding perovskite‐type material, SrCe_0.95_Yb_0.05_O_3‐α_, are one of the contenders for good proton conductors of hydrogen pumping systems that have been explored. **Table** **5** represents the fabrication method, sintering temperature, gas mixture, tritium activation energy for conduction, and tritium conductivity of a cerate‐based tritium recovery system.^[^ ^187^ ^]^

**Table 5:** Cerate‐based tritium recovery system with fabrication method, sintering temperature, gas mixture, tritium activation energy, and tritium conductivity.

Electrolyte materials	Fabrication method	Sintering temperature/time	Titrium activation energy, ΔH_T_	Titrium conductivity, σ_T_	Gas mixture	Refs.
BaCe_0.9_Yb_0.1_O_2.95_	Solid state	1923 K for 10 h	0.56 eV	1 mS cm^−1^ at 600 °C	2% T_2_/argon	[187]
SrZr_0.9_Yb_0.1_O_2.95_	Solid state	1923 K for 10 h	0.61 eV	0.7 mS cm^−1^ at 600 °C	2% T_2_/argon	

### 4.4 Performance Analysis of Cerate Based Hydrogen Sensors

Borland et al. reported the different synthesized techniques and the performance of BaZr_0.9_Y_0.1_O_3_, BaCe_0.6_Zr_0.3_Y_0.1_O_3‐δ_, Sr(Ce_0.6_‐Zr_0.4_)_0.9_Y_0.1_O_3‐δ_, and Sr_3_Fe_1.8_Co_0.2_O_7‐δ_ proton conductors as electrolytes in a hydrogen sensor.^[^ ^165^ ^]^ The solid‐state reaction method has been preferred to synthesize BaZr_0.9_Y_0.1_O_3_ and BaCe_0.6_Zr_0.3_Y_0.1_O_3‐δ_ proton conducting membranes, while the citrate method has been used for the synthesis of Sr(Ce_0.6_‐Zr_0.4_)_0.9_Y_0.1_O_3‐δ_ and Sr_3_Fe_1.8_Co_0.2_O_7‐δ_ proton conducting membranes. Hydrogen sensors synthesized using BaCe_0.6_Zr_0.3_Y_0.1_O_3‐δ_ and Sr_3_Fe_1.8_Co_0.2_O_7‐δ_ proton exchange membranes exhibit the best performances with stable output potentials having minimal ≈50 mV deviation from the theoretical value obtained from the Nernst relation. A slightly higher (≈76 mV) potential deviation is observed from BaZr_0.9_Y_0.1_O_3,_ which may be attributed to noisy signals from the experimental setup. However, Sr(Ce_0.6_‐Zr_0.4_)_0.9_Y_0.1_O_3‐δ_ perovskite ceramic does not exhibit significant promise as a proton exchange membrane. Still, it is helpful for electrochemical sensing owing to the enormous potential deviations between the observed and the theoretical values.^[^ ^196^ ^]^ **Table** **6** demonstrates the cerate‐based hydrogen sensor (Part‐1) with the synthesis process and different parameters, including thickness, density, electrode, proton conductivity, temperature, atmosphere, and hydrogen concentration.^[^ ^166^, ^169^ ^]^ Cerate‐based hydrogen sensor (Part‐2) with synthesis process, electrode, working temperature, theoretical and experimental electrochemical potential, and deviations are shown in **Table** **7**.^[^ ^165^ ^]^

**Table 6:** Cerated‐based hydrogen sensor (Part‐1).

Electrolyte materials	Synthesis method	Thick. [mm]/Dia. [mm]	Density	Electrode	Proton conductivity [mS cm^−1^]	Temperature [°C]	Atmosphere	Hydrogen concentration/Temperature [°C]	Refs.
BaCe_0.7_Zr_0.1_Y_0.2_O_3− δ_	citrate‐nitrate combustion	1/16	Highly dense (95%)	Pt/Pt	0.31	350	N_2_ + 2% H_2_O + 7% H_2_	0.1–10 vol.%/450–550 °C	[169]
					15.1	750	N_2_ + 2% H_2_O + 7% H_2_	0.1–10 vol.%/450–550 °C	
BaZr_0.4_Ce_0.4_In_0.2_O_3_	solid‐state reaction	0.5/13	In excess of 96%	Pt/Pt	20	800	Wet Hydrogen	0–100% at each temp.	[166]
					3	500	Wet Hydrogen		

**Table 7:** Cerate‐based hydrogen sensor (Part‐2).

Electrolyte materials	Synthesis method	Electrode/area [cm^2^]	Working temperature [°C]	Theoretical electrochemical potential [mV]	Experimental electrochemical potential [mV]	Deviations [mV]	Refs.
BaCe_0.6_Zr_0.3_Y_0.1_O_3‐δ_	solid‐state reaction	Pt/0.5	500	−176	−125	51	[165]
Sr(Ce_0.6_‐Zr_0.4_)_0.9_Y_0.1_O_3‐δ_	citrate method	Pt/0.5	500	−176	−25	146	

Furthermore, Borland et al. designed a lab‐scale hydrogen sensor unit and injected two different gas mixtures (Ar and H_2_) of known H_2_ partial pressure into both compartments. Knowing the theoretical electrochemical cell potential (−176 mV), the authors observed the deviation in electrochemical cell potential for different proton conductors at 500 ⁰C, 0.0001 bar P_H2, WE,_ and 0.02 bar P_H2, RE_. Although the observed electrochemical cell potential for BaZrY (−100 mV) and BaCeZrY (−120 mV) was stable and very close to the theoretical value (−176 mV), the electrochemical cell potential for SrCeZrY (−30 mV) showed significant deviation. Also, it required comparatively more time to yield a stable cell potential. Any modification in the structural composition of perovskites with dopants changes the activation limit of oxygen vacancy formation; hence, it is crucial to investigate the deviation of the electrochemical cell potential of the perovskite candidates within a series of elevated temperatures (600–1000 ⁰C).^[^ ^197^ ^]^ Kalyakin et al. designed a sensor using BaCe_0.7_Zr_0.1_Y_0.2_O_3‐δ_ as cell material and tested the electrochemical properties in different operating conditions (H_2_ content and temperature).^[^ ^169^ ^]^ BaCe_0.7_Zr_0.1_Y_0.2_O_3‐δ_ experienced an increase in ionic conductivity primarily at ≈620 ⁰C due to their phase transition toward a higher symmetric structure and dissociation of weak bonds between point defects such as ${\left\{V_{O}^{.}-Y_{Ce,\;Zr}^{\prime }\right\}}^{\cdot }$ and ${\left\{OH_{O}^{\;.}-Y_{Ce,\;Zr}^{\prime }\right\}}^{x}$. For this reason, BaCe_0.7_Zr_0.1_Y_0.2_O_3‐δ_ materials could become more efficient proton conductors between 450 and 550 ⁰C, as demonstrated in their proton conductivity test. This study also examines the BaCe_0.7_Zr_0.1_Y_0.2_O_3‐δ_ as an electrolyte for a hydrogen sensor that operates in both potentiometric and amperometric modes.

**Figure 12:**
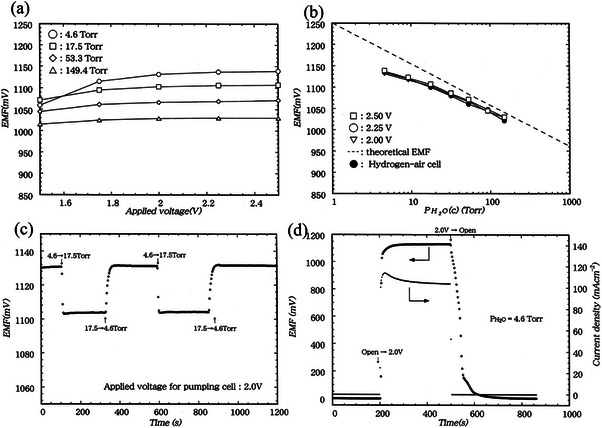
At 700 °C, a) water vapor pressure in the test gas (air) is measured as a function of the change in the sensing cell's EMF when voltage is delivered to the pumping cell. b) EMF response of the sensor to air. c) A steam sensor's response to an EMF. d) Applying 2.0 V to the pumping cell causes the sensor's EMF and current to change. Reproduced with permission from ref. [199].

Co‐doping Indium was introduced into BZC composites, as such composites have better stability under steam conditions.^[^ ^198^ ^]^ A detailed study of their conduction behavior is reported by N. Taniguchi et al. (2005).^[^ ^166^ ^]^ The conduction properties of BZCI were investigated based on the cell's EMF response to hydrogen concentration, and electrochemical H_2_ permeation was carried out. Their study reveals that BZCI ceramic showed good proton conduction properties at 300–700 °C in the presence of hydrogen, and as a potential application in limiting current‐type hydrogen sensors, exhibits good sensing characteristics in a reducing atmosphere. Furthermore, the cyclic study (100 cycles) revealed that the material has robust mechanical stability. In addition, the impact of adding contaminant gases like CH_4_, C_3_H_8_, and C_4_H_10_ in hydrogen sensors with BZCI ceramics was observed. **Figure** **12**a depicts the relationship between the sensing cell's EMF and the voltage applied to the pumping cell while different vapor pressures are considered.^[^ ^199^ ^]^ At voltages exceeding 2.0 V above the applied voltage, a constant EMF was seen for each water vapor pressure in the test gas. Figure 12b represents the EMF responses of the sensor to air with different pumping voltages. The measured EMF was a monotone logarithmic function of $P_{H_{2}O}$ and was unaffected by applied voltages greater than 2.0 V. A plot of the sensing cell's EMF and the pumping cell's current density versus the voltage supplied to the pumping cell is shown in Figure 12c,d; the water vapor pressure in the test gas was 4.6 Torr. Current density started to increase at ≈1.25 V when the pumping cell was exposed to the applied voltage. At this voltage, the EMF of the sensor cell increased sharply and nearly stabilized.^[^ ^199^ ^]^

## 5 Challenges and Prospects of Cerate Material in Electrochemical Hydrogen Devices

Cerate‐based PCOs used as electrolytes in electrochemical devices face challenges, including proton conductivity, stability, doping concentration, and temperature. In the following sub‐section, the challenges and prospects of cerate‐based PCOs used as an electrolyte in electrochemical devices are discussed and presented.

### 5.1 Challenges and Limitations of Cerate Materials in Electrochemical Hydrogen Devices

The challenges and limitations of cerate material‐based electrochemical hydrogen devices are discussed in the following categories.

#### 5.1.1 Challenges and Limitations of Cerate Materials in Hydrogen Pumps

BaCeO_3_ and SrCeO_3_ are the most promising proton‐conducting materials due to their excellent proton conductivity, which can be used as electrolytes in a hydrogen pump system. One of the challenges associated with these PCOs is that they interact with steam water and acidic gases and therefore have poor stability. Although each element must overcome unique material disadvantages, the interesting trend suggests that doping may be a common strategy to overcome these challenges. The hydrogen pump using doped SrCe_0.95_Y_0.05_O_3‐α_ as an electrolyte showed poor oxide ionic conductivity and higher proton conductivity with decreased temperature because water vapor could be reduced to H_2_ at the cathode at low temperatures.^[^ ^176^ ^]^ As a result, electronic charges within the electrolyte were restrained.^[^ ^200^ ^]^ However, the decrease in operating temperature costs more voltage to maintain the same pumping current. As suggested by the authors, the above challenges could be overcome by reducing the thickness of the electrolyte and with moisture in the carrier gas. Another challenge is that the durability and stability of SrCe_0.95_Yb_0.05_O_3‐α_ remain doubtful owing to its reaction with increased CO_2_ in the system following the application of a current. Although possessing a high protonic conductivity, poor electronic conductivity could make this material inappropriate for use in hydrogen pumps with mixed ionic and electronic conduction.^[^ ^201^ ^]^ However, these proton‐conducting electrolytes are more advantageous than oxide ion‐conducting electrolytes owing to their high protonic conductivity and low activation energy, making them useful for hydrogen pump systems and electrolysis operations.^[^ ^202^ ^]^

Recently, Galvanic ceramic hydrogen pumps have attracted a lot of attention due to their complete solid‐state design and capacity to function in harsh environments, as reported by Iwahara et al. in their research on the extraction of H_2_ from the gas mixture using SrCe_0.95_Yb_0.05_O_3‐α_ at 800–900 °C.^[^ ^203^ ^]^ The challenges are that both the interfacial polarizations of electrodes and the ohmic resistances of electrolytes need to be decreased to increase the efficiency of hydrogen pumping. This problem can be overcome by thinning the electrolyte or using novel electrolytes with better protonic conductivities like BaZr_0.7_Ce_0.2_Y_0.1_O_3‐α_; the pure ohmic resistances can be decreased.^[^ ^178^ ^]^ However, the hydrogen pumping properties are also influenced by operational factors such as temperature and the partial pressures of hydrogen and water vapor in the mixture.^[^ ^74^, ^88^ ^]^ The current ceramic hydrogen pumps are primarily used in high‐temperature applications between 700 and 900 °C, which presents considerable challenges for cost‐competitiveness, dependability, and durability over the long term. To overcome this problem, Tong et al. designed a tri‐layered hydrogen pump using BaZr_0.1_Ce_0.7_Y_0.2_O_3‐δ_as an electrolyte, with high hydrogen pumping performance at the lower operating temperature range of 350–500 °C where the interfacial polarization resistances of electrodes and the ohmic resistances of electrolytes were low.^[^ ^140^ ^]^

As we know, BaZrO_3_ exhibited the highest chemical stability compared to other PCOs, and BaCeO_3_ offered the highest proton conductivity. Therefore, to optimize the properties of PCOs, it has become popular recently to mix these two perovskite PCOs.^[^ ^167^ ^]^ Depending on the desired outcome, Zr or Ce can be incorporated into BaZrO_3_ or BaCeO_3_. Like adding Ce to BaZrO_3_ increases its overall conductivity, sintering reduces the material's chemical stability in the presence of CO_2_ and H_2_O. The synthesis routes are important in addition to the final chemical makeup of the composite. However, compared to the wet chemical and citrate combustion approach, the solid‐state reaction method was said to create composites with improved performance.^[^ ^204^ ^]^ Doping rare earth elements with mixed BaCeO_3‐_BaZrO_3_ perovskite oxides improves the properties of the composites. Therefore, more research still needs to be done to optimize the material properties.

Hydrogen pumps that use BaCeO₃‐based electrolytes face challenges during sintering and need very high processing temperatures (usually above 1400 °C) to become dense enough. This can cause unwanted phase formation and higher costs, but using sintering aids like ZnO or advanced techniques like spark plasma sintering can help lower the required temperature.^[^ ^205^ ^]^ The long‐term stability in humid, CO₂‐rich environments is weakened by the formation of harmful carbonates like BaCO₃, which reduce proton conductivity and structural strength. To fix this, replacing some Ce⁴⁺ with Zr⁴⁺ in BaZr_x_Ce₁₋_x_O₃_−δ_ electrolytes improves chemical stability without much loss in conductivity.^[^ ^206^ ^]^ The compatibility with electrodes and interconnects is an issue because of mismatched thermal expansion and reactive interfaces, which create unstable or insulating phases. This problem can be solved by using electrode materials like Ni‐based cermets and adding buffer layers to maintain stable interfaces.^[^ ^207^ ^]^

#### 5.1.2 Challenges and Limitations of Cerate Materials in Hydrogen Isotope Separation System

Using proton‐conducting materials for separating hydrogen isotopes depends on slight variations in the mobility of deuterium (D) and hydrogen (H) ions across a solid electrolyte.^[^ ^126^ ^]^ The transport of hydrogen isotopes in the form of protons (H^+^) and deuterons (D^+^) under an applied electric field or chemical potential is facilitated by proton‐conductors, which are usually ceramic oxides or polymer membranes.^[^ ^208^ ^]^ H^+^ ions are more mobile and have lower activation energy for conduction than D^+^ ions because of their different masses.^[^ ^138^ ^]^ Protium preferentially transports over deuterium due to its mass‐dependent mobility, allowing for their separation. A variety of factors, including temperature, material composition, microstructure, and the isotope effect in proton conduction, affect how effective the separation process is.^[^ ^208^ ^]^

Proton‐conducting ceramics such as BaCeO_3_ and SrCeO_3_ have extremely high melting points, 2016 and 2266 K.^[^ ^209^ ^]^ Due to these high melting points, developing the qualifying (or gastight) membranes may be challenging. Above mentioned ceramics must be sintered at a very high temperature to attain the appropriate densification. However, lowering the sintering temperature could be possible by using modest amounts of sintering aids such as ZnO, Co_2_O_3_, CuO, etc.^[^ ^210^ ^]^ Another challenge is that proton‐conducting cerate‐based perovskite, as a hydrogen separation membrane, demonstrated poor hydrogen flux compared to the metal additive‐based membrane. The poor hydrogen flux hinders the application of proton‐conducting cerate in the device applications, even in the doped oxide systems.^[^ ^211^ ^]^ For example, the flux of permeated hydrogen lowered through the cobalt‐doped BaCe_0.85_Tb_0.05_Co_0.1_O_3‐δ_ membrane compared to the undoped one, despite exhibiting higher electrical conductivity.^[^ ^183^ ^]^ This may be due to the larger thickness of the doped BaCe_0.85_Tb_0.05_Co_0.1_O_3‐δ_ fibers and the underdeveloped inner surface porous layer. On the other hand, the PCOs, Ni‐BZCY (Ni–BaZr_0.1_Ce_0.7_Y_0.2_O_3−_ *_δ_*), exhibited low stability in the case of H_2_S at 900 °C, immediately hindering the overall hydrogen permeability.^[^ ^184^ ^]^ To overcome these challenges, the improvement of H_2_S tolerance with the increase of partial pressure of water in the feed is necessary for the potential application of the BaZr_0.1_Ce_0.7_Y_0.2_O_3‐δ_ cermet membrane.

Two major challenges with the conventional disk‐shaped membrane of perovskite oxides for H_2_ separation are their low permeability (0.001 mL cm^−2^ min^−1^) and high sintering temperature (above 1400 °C) to reach the desired densification for the gas‐tight environment. At such elevated temperatures, the atomic position of the A site and B site cation disrupts the structural phase, and consequently, the material's overall performance is drastically reduced. To overcome these challenges, Tan et al. synthesized BaCe_0.8_Y_0.2_O_3−_ *_δ_* hollow membranes with the addition of 1 weight% % sintering aid (Co_2_O_3_) by following phase inversion and sintering techniques.^[^ ^183^ ^]^ The authors showed that the sintering aid reduced the need for sintering temperature to reach the desired mechanical strength for a gas‐tight environment by 200 °C.

EHPs have been developed using various ceramic proton‐conducting membranes where hydrogen separation is carried out through the application of a potential difference across the electrolyte. However, such a galvanic system (hydrogen pump) is often inconvenient and cost‐inefficient owing to the high consumption of electrical energy. A nongalvanic system using proton‐conducting membranes for hydrogen separation is prospective in this regard due to no electrodes being used in this system. As a result, no power is consumed.^[^ ^212^ ^]^ The thickness of the proton‐conducting membrane plays an important part in this context since hydrogen permeation through the membrane is inversely proportional to its thickness. Therefore, the use of thin films of ceramic proton‐conducting membranes for hydrogen separation has gathered much attention. Moreover, the use of thin‐film electrolytes has the added advantages of reduced operating temperatures and minimized loss of electrical energy. However, the synthesis of high proton‐conducting thin film electrolytes is difficult. The sintering technique often requires high operating temperatures that can hamper thermal stability, inducing a higher cost of operation and complex processing steps.^[^ ^213^ ^]^ Apart from this, without incorporating a reference electrode in the system, the electrode overpotential contributions to the applied voltage were not measured and reported. Furthermore, the oxide powder reaction to fabricate protonic conductor‐based Ni cermet is often complex and expensive, which may also generate inhomogeneous results.^[^ ^214^ ^]^ Therefore, further research is required to resolve these challenges.

Hydrogen isotope separation using cerate‐based electrochemical systems faces several challenges. One of the primary issues is that cerate materials have low protonic conductivity at intermediate temperatures, limiting the effectiveness of isotope separation processes. Furthermore, cerate materials' chemical stability in humid conditions is a concern because they have a tendency to degrade, which affects the separation systems' longevity and performance.^[^ ^126^ ^]^ In addition, the technological difficulty of fabricating dense, flawless cerate membranes limits the devices' scalability for industrial use.^[^ ^215^ ^]^ In order to overcome these challenges, researchers are investigating the development of dual‐phase membranes, which integrate cerate materials with electronically conductive phases to improve overall stability and conductivity. Additionally, improvements in fabrication methods, such as sintering aids and improved processing conditions, are being investigated to develop superior cerate membranes that are appropriate for effective hydrogen isotope separation.^[^ ^216^ ^]^

Cerate‐based electrochemical hydrogen isotope separation systems also suffer from undesirable interphase reactions, compromising membrane integrity and performance, due to the high sintering temperatures required for densifying refractory cerate electrolytes. Dual‐phase reaction sintering techniques have been developed to address this issue. These techniques reduce inter‐phase interactions and manufacturing costs by achieving densification at lower temperatures using a combination of fast and slow sintering phases.^[^ ^217^ ^]^ Long‐term stability in humid CO₂‐rich environments is also an issue, as carbonates can be formed when CO₂ reacts with cerate materials, which degrade membrane performance. A recent study has demonstrated that co‐doping cerates with Zr and Yb improves chemical stability and preserves hydrogen permeability even in the presence of moist CO₂. Additionally, connection and electrode compatibility are crucial to prevent interfacial interactions that might hamper device performance. Sintering aids like ZnO have been successfully used to reduce interfacial reactions and lower sintering temperatures, improving membrane performance and compatibility.^[^ ^216^ ^]^

#### 5.1.3 Challenges and Limitations of Cerate Materials in Tritium Recovery Systems

Ceramic proton conductors such as SrCe_0.95_Yb_0.05_O_3‐α_ are used for a tritium recovery system with low electric conductivity, making locating carbon on the ceramic surface challenging.^[^ ^191^ ^]^ The CH_4_+H_2_O|Ni|SrCe_0.95_Yb_0.05_O_3‐α_|NiO|O_2_+H_2_O system is especially useful since it allows for the relatively simple recovery of oxidized tritium as tritium water vapor in the anode side. However, the lower protonic conductivity of ceramics such as SrCe_0.95_Yb_0.05_O_3‐α_ is a concern and may result in a lower hydrogen evolution rate. To prevent the reduction of proton conductivity, increasing the oxygen partial pressure on the cathode side could be a solution to increase both electrical resistance and proton conductivity. Other challenges involved with the use of membrane permeation, such as Pd‐Ag, especially in systems involving low tritium partial pressure, incorporating water vapor processing.^[^ ^188^, ^189^ ^]^ However, the transport properties of hydrogen isotopes for He sweeps gas containing multiple components such as H_2_, HT, HTO, and H_2_O have not been studied extensively for the tritium recovery system. Apart from hydrogen pumping, the system's viability for CH_4_ decomposition is another concern, and the rate‐determining step is not established either. Although such a system exhibits promising prospects, extracting pure hydrogen isotopes would require system operation in a vacuum environment to avoid purge gas contamination.^[^ ^218^ ^]^

Studying whether high vacuum conditions could further improve the hydrogen recovery rate is also necessary. A large surface area of the electrode is also needed in the application of tube‐type proton‐conducting oxide to boost the current density. Otherwise, the number of tubes needed would rise, possibly increasing the cost. However, proton‐conducting SrCe_0.95_Yb_0.05_O_3‐α_ ceramic tubes have been utilized to design hydrogen pumps that can extract hydrogen isotopes from He sweeps gas for an H_2_–H_2_O gas mixture in a blanket tritium recovery system.^[^ ^162^ ^]^ Some research groups studied hydrogen isotope transport properties and demonstrated that hydrogen isotopes are split up, absorbed by the electrode, and then diffused via the lattice structure.^[^ ^177^ ^]^ However, the tritium concentration is observed to be very low compared to that of hydrogen, owing to the high volumes of hydrogen transportation in the system. Moreover, though H_2_O vapor is not fed to this electrochemical pump while extracting the hydrogen isotopes, traces of H_2_O vapor present in the outlet gas were detected.^[^ ^162^ ^]^ This could be because hydrogen isotopes, after permeation, are oxidized and then form H_2_O vapor in the permeation site.^[^ ^218^ ^]^ Hence, extensive research is needed on the hydrogen isotope extraction and compatibility with cerate electrolytes.

Tritium recovery systems that use electrochemical hydrogen devices based on cerate face several major challenges. One of the primary challenges is the high operating temperatures required for cerate materials, including CaZr₀.₉₁In₀.₁O₃_−α_, which performs effectively at 873–1073 K. This temperature range can lower the evolution rates of hydrogen and tritium by reducing proton solubility and electrode reaction kinetics. To mitigate this, researchers suggest increasing the electrode area to enhance current application, aiming to improve hydrogen recovery rates.^[^ ^56^ ^]^ Another major challenge is that solid polymer electrolytes (SPEs) like Nafion can degrade under high radiation from tritiated water, affecting performance. To overcome this, more stable alternatives such as montmorillonite–kaolin composite membranes have been developed, offering better durability and hydrogen isotope separation. Electrolysis provides high separation efficiency but is energy‐intensive and costly on its own for large‐scale use. Combining electrolysis with catalytic exchange, as in the CECE process, improves efficiency while lowering energy demands.^[^ ^219^ ^]^ Material degradation from tritium exposure is a major concern. ZrCo alloys, often used for tritium storage, require high activation energy, which limits their efficiency. Adding manganese to these alloys helps improve activation and hydrogen absorption. Although MXene lamellar membranes show potential for accurate hydrogen isotope separation, making them and understanding how molecules move through their tiny channels are still challenging.^[^ ^220^ ^]^

Tritium recovery systems in cerate‐based electrochemical hydrogen devices suffer from sintering difficulties and high processing temperatures. Sintering at elevated temperatures can lead to grain growth and phase instability, adversely affecting proton conductivity. To address this, recent studies have explored nanostructured ceramic membranes that operate effectively at lower temperatures, enhancing the efficiency of hydrogen isotope separation without compromising structural integrity.^[^ ^163^ ^]^ Cerate‐based materials can be damaged when exposed to humid and CO₂‐rich environments, as carbonation and hydration reduce their proton conductivity and cause degradation. Studies suggest that changing the ceramic composition and using protective coatings can help reduce these effects, improving the material's durability in fusion reactors. Additionally, reactions between the ceramics and electrodes or interconnects can harm performance. Using composite electrodes, like those with cerium oxide, has been found to improve electrolysis efficiency and make the materials more compatible with cerate ceramics, boosting the overall performance of tritium recovery systems.^[^ ^221^ ^]^

#### 5.1.4 Challenges and Limitations of Cerate Materials in Hydrogen Sensors

Despite the many benefits, there are still some difficulties when employing cerate‐based PCOs for hydrogen sensors. The performance of the hydrogen sensor depends on the proton conductivity of PCOs. Undoped cerate‐based PCOs exhibited lower proton conductivity and chemical stability in the harsh atmosphere, hence, doped cerate‐based PCOs are important for hydrogen sensors. For example, doped rare earth elements with cerate, such as SrCe_0.95_Yb_0.05_O_3‐α,_ showed higher proton conductivity and stability in CO_2_ and H_2_O vapor environments.^[^ ^199^ ^]^ The current‐type hydrogen sensor, which has a proton conductor with good chemical stability and proton conductivity, is capable of limiting current.^[^ ^166^ ^]^ According to recent investigations, the limiting current in the hydrogen sensor increased as the voltage across the electrode increased, and the partial pressure also relates to the limiting current.^[^ ^222^ ^]^ The hydrogen concentration determines the limiting current linearly, and diffusion control can keep it stable. However, when SrCe_0.95_Yb_0.05_O_3‐α_ is used as the solid electrolyte in a steam sensor, it has shown a constant EMF for each water vapor pressure in the test gas above 2.0 V of the applied voltage.^[^ ^199^ ^]^ However, the EMF value is slightly smaller than the theoretical calculation, which might be due to partial hole conduction appearing in the electrolyte. This hole conduction increased with decreasing partial pressure of steam, so the sensing capability of this setup at lower partial pressure of steam needs to be analyzed further. The temperature of the experiment was 700 °C, and the behavior at a higher temperature needs to be analyzed.

In the case of BaZr_0.4_Ce_0.4_In_0.2_O_3_ (BZCI), despite the protons being the charge carriers, electronic conduction occurs at temperatures above 800 °C. As a result, the EMF values of the sensory compartment were below theoretical values above this temperature. The proton conduction increases with decreasing temperature in BZCI in the absence of oxygen. Further analysis on whether the same phenomenon occurs in the presence of oxygen has to be carried out to increase the applicability of this electrolyte material.^[^ ^166^ ^]^ For the application of the sensors, excellent microstructure properties, i.e., free from any pores, are essential for the sintered material. Otherwise, uncontrolled gas leakage can occur through the pores, resulting in poor electrochemical responses due to side reactions. Furthermore, expansion characteristics are critical for evaluating the performance of the materials in high‐temperature devices, which are yet to be determined for many cerates. Again, at high temperatures, phase transitions of BaCeO_3_‐based materials occur, resulting in different thermal expansion values. Barium cerate and barium zirconates exhibited high ionic conductivity and coexisted with ionic‐electronic conductivity in an oxidizing environment. Also, the oxygen‐ionic transport predominates at high temperatures, whereas the proton transport is accomplished at lower temperatures and in steam atmospheres (H_2_O).^[^ ^169^ ^]^ However, doping with cerate‐based PCOs still needs more research to optimize the hydrogen sensor performance. In addition, most hydrogen sensors are operated at high temperatures, yet the sensor's performance at high temperatures has not been evaluated. Therefore, Future researchers can continue to explore hydrogen sensor performance at high temperatures.

Hydrogen sensors based on cerate materials, such as barium cerate (BaCeO₃), degrade into CeO₂ and Ba(OH)₂ below 900 °C, making them ineffective in humid and CO₂‐rich conditions. This lowers the reliability and long‐term effectiveness of the sensors. The decomposition rate is significantly higher for doped variants, such as BaCe₀.₈Gd₀.₂O₃. As a way to address this issue, researchers are conducting research with alternatives such as yttrium‐doped barium zirconate (BaZrO₃), which provides improved stability and proton conductivity in challenging circumstances.^[^ ^223^ ^]^ Another challenge is the necessity of valuable metals, such as platinum, for electrode materials, which raises expenses and may result in issues such as particle aggregation, which lowers active surface area and sensor effectiveness. A possible solution is to develop noble‐metal‐free sensors with mixed ionic–electronic conductors (MIECs), like SrFe₀.₅Ti₀.₅O₃₋δ, which have demonstrated superior stability, sensitivity, and selectivity without requiring costly metals.^[^ ^224^, ^225^ ^]^ Furthermore, humidity and cross‐sensitivity to other gases may hamper the function of cerate‐based sensors, resulting in inaccurate results. In order to mitigate this, researchers are investigating surface functionalization approaches and nanostructured materials to improve selectivity and limit interference from other gases.^[^ ^226^, ^227^ ^]^

Cerate‐based electrochemical hydrogen sensors can lead to grain growth and porosity issues, adversely affecting their conductivity and mechanical strength, due to the high sintering temperatures. For instance, BaCe₀.₆Zr₀.₃Y₀.₁O₃‐α ceramics typically necessitate sintering at temperatures ≈1400 °C to achieve desired densities. This can be minimized by using sintering aids like ZnO, which have been demonstrated to improve densification and lower the required sintering temperature, thereby improving the material's performance and microstructure.^[^ ^130^, ^228^ ^]^ The long‐term stability of cerate‐based sensors in humid and CO₂‐rich environments is also a significant concern. Materials based on BaCeO₃ are subjected to decomposition under such conditions, resulting in the formation of BaCO₃ and CeO₂, which reduces their proton conductivity. Chemical stability has been effectively improved by substituting zirconium or other more stable elements with cerium. For example, BaZr₀.₈Y₀.₂O₃_‐δ_ has better stability against CO₂ and moisture, which makes it more suitable for long‐term use. Additionally, sensor efficiency depends on compatibility with electrodes and interconnects. Inconsistencies in chemical reactivity and thermal expansion coefficients may cause delamination or degradation at the interfaces. Interfacial stability and overall sensor performance can be improved by using compatible electrode materials, such as platinum or modified perovskite oxides, and making sure that their thermal properties match.^[^ ^223^, ^229^ ^]^

### 5.2 Prospects of Cerate Materials in Electrochemical Hydrogen Devices

The potential of cerate‐based materials, particularly PCOs, in electrochemical hydrogen devices, is drawing attention because of their superior electrochemical properties and outstanding proton conductivity. These materials are being explored for application in energy conversion systems, sensors, and hydrogen pumps. Recent studies have demonstrated the effectiveness of PCECs utilizing cerate‐based electrolytes for efficient hydrogen separation. For instance, a novel PCEC was reported to achieve Faraday efficiencies exceeding 96% when properly separating pure hydrogen from diluted streams at temperatures ranging from 350 to 500 °C. The well‐bonded electrochemical interfaces and nanoporous nickel catalysts produced by carefully controlled in situ reduction of nickel oxides are responsible for this performance.^[^ ^230^ ^]^ PCECs using cerate‐based materials have demonstrated remarkable stability and reversibility in terms of producing energy and hydrogen. According to a study, researchers used a cerate‐based electrolyte, which conducts protons extremely well, in combination with a unique air electrode that allows protons to flow through. This configuration had minimal performance loss over a long period of time and operated effectively in both fuel cell and electrolysis modes.^[^ ^231^ ^]^ Improvements in material synthesis have also helped cerate‐based electrochemical devices perform better. For example, using the molten salt method improved the hydrogen evolution reaction (HER) performance in both acidic and COS electrolytes for cerium‐modified rhenium disulfide (Ce‐ReS₂). The inclusion of cerium accelerated the dynamics of reactions and enhanced the number of active sites, providing insights into the development of effective electrocatalysts.^[^ ^232^ ^]^

The commercialization readiness of cerate material‐based electrochemical hydrogen devices is advancing, with notable progress in PCECs utilizing cerate‐based electrolytes. These devices perform smoothly in steam electrolysis at intermediate temperatures (500–650 °C) and demonstrate remarkable reversibility, stability, and Faradaic efficiency. These systems are suitable for scalable hydrogen production as the incorporation of cerate materials improves their longevity and performance. In addition, the advancement of electrochemical hydrogen compressors (EHCs) provides a compact and efficient approach to purifying and compressing hydrogen, resolving issues with traditional techniques. These advancements indicate a significant step toward the commercialization of cerate material‐based electrochemical hydrogen devices, paving the way for their integration into sustainable energy systems.^[^ ^231^, ^233^ ^]^ Recent developments demonstrate the potential of these technologies, such as Ceres Power's development of a 1 MW solid oxide electrolyzer. This electrolyzer produces one kilogram of hydrogen with less than 40 kWh of electricity. At Shell's R&D Center in Bangalore, it is currently being prepared for use for further testing and is up to 25% more efficient than the current technology.^[^ ^234^ ^]^ Researchers have developed PCECs utilizing cerate‐based materials, specifically employing BaZr₀.₄Ce₀.₄Y₀.₁Yb₀.₁O₃ (BZCYYb4411) as the electrolyte. These cells demonstrate remarkable durability and reversibility, maintaining performance for 500 h at 550 °C and achieving a 76% Faradaic efficiency. These developments demonstrate the potential of cerate‐based PCECs in producing power and hydrogen efficiently.^[^ ^231^ ^]^

Cerate‐based electrochemical hydrogen devices, especially those utilizing proton‐conducting ceramics such as BaZr₀.₄Ce₀.₄Y₀.₂O₃ (BZCY), offer attractive hydrogen production possibilities because of their strong ionic conductivity and ability to operate at intermediate temperatures (500–700 °C). Significant improvements in current densities have been demonstrated in recent developments; values as high as −1.92 A cm^−^^2^ at 1.3 V and 600 °C indicate improved performance in steam electrolysis applications.^[^ ^231^, ^235^ ^]^ In comparison, PEM electrolyzers function at lower temperatures (20–80 °C) and are distinguished by their high‐purity hydrogen output, rapid startup, and compact construction. However, they are sensitive to water purity and frequently need costly noble metal catalysts, which might affect their longevity and long‐term operating costs. Additionally, hybrid systems attempt to find a balance between operating flexibility and efficiency by integrating elements of both low‐temperature and high‐temperature technology. However, they have difficulties with system complexity and material compatibility. Overall, even though cerate‐based devices are still in the early stages of development, they present a viable alternative to current hydrogen production technologies due to their potential for high efficiency and operating at intermediate temperatures.^[^ ^236^, ^237^ ^]^

Proton‐conducting materials such as barium cerate (BaCeO₃) are used in cerate‐based electrochemical hydrogen devices. These devices are becoming more important in fusion reactors and hydrogen systems because they work efficiently and reliably.^[^ ^56^ ^]^ These materials perform well at intermediate temperatures (350–600 °C), which are aligned with the thermal profiles of nuclear fission and fusion reactors, allowing for the effective production of hydrogen and the generation of energy. Such compatibility facilitates the integration of these devices into existing energy systems, enhancing overall efficiency.^[^ ^238^ ^]^ In fusion reactors, hydrogen pumps using proton‐conducting ceramics are being studied for important tasks like separating hydrogen isotopes and recovering tritium. These functions help keep the reactor safe and make it more cost‐effective.^[^ ^239^ ^]^ In addition, developments in pressurized protonic ceramic electrolyzers have shown enhanced electrode activity and lower power needs for water electrolysis, which makes them suitable for fusion energy systems and scalable hydrogen production. These developments highlight the critical role of cerate‐based electrochemical devices in developing the hydrogen economy as well as fusion energy applications.^[^ ^240^ ^]^

## 6 Conclusions and Recommendations for Future Work

The growing demand for clean energy, the global concern for environmental issues, and the depletion of fossil fuels insist that the scientific community find an environmentally friendly alternative. Electrochemical hydrogen technology has demonstrated significant potential as a clean and sustainable energy source. The performance of electrochemical hydrogen devices depends on highly conductive and stable PCOs that are used as an electrolyte to transport protons. Cerate‐based PCOs like BaCeO_3_ and SrCeO_3_ are particularly promising candidates compared to other PCO materials. This is due to their exceptional ability to transport protons and their inherent stability arising from specific grain boundary properties and structural characteristics. Therefore, BaCeO_3_ and SrCeO_3_ are attractive materials for an electrolyte in electrochemical device applications. However, the stability of BaCeO_3_ is low in CO_2_ and H_2_O vapor atmospheres, even though they exhibit the highest proton conductivity. However, doped BaCeO_3_ and SrCeO_3_ with metallic and nonmetallic materials improved the chemical stability and proton transport properties. In addition, the cerate‐zirconate‐based perovskite showed excellent chemical stability with proton conductivity in a harsh atmosphere. Doping trivalent ions into the BaCeZrYO_3_ (BCZY) electrolyte will increase its chemical stability and ionic conductivity.

The sintering aid, electrode type, and synthesis procedures are also essential to improve the material manufacturing process, stability, and proton transport capabilities. Additional development and modification are required to optimize the performance of the electrolytes at a suitable temperature. This study highlights several studies on cerate‐based materials used as electrolytes in hydrogen pumps, hydrogen isotope separation systems, tritium recovery systems, and hydrogen sensors. It is noteworthy that all the implemented techniques, synthesis processes, and adjustments, while aiming to enhance electrolyte stability and proton transport capability, can also lead to a varying degree of compromise in structural integrity. This trade‐off remains a significant hurdle for the commercial viability of these materials. In this paper, we have also clarified the challenges and prospects of cerate materials, which provide prospective researchers with a path to make a significant impact in this field. We can draw the following conclusions summarizing some key findings about the cerate PCO in hydrogen devices.

i. BaZr_0.8_Ce_0.1_Y_0.1_O_3‐δ_ based galvanic devices have shown superior hydrogen pumping efficiency. Other cerate materials like BaZr_0.1_Ce_0.7_Y_0.2_O_3‐δ,_ Ba(Zr_0⋅85_Y_0.15_)O_3‐δ_‐(La_0⋅7_Sr_0⋅3_), V_0.9_O_3‐δ_@CeO_2_, Pd|Ba(Zr_0.30_Ce_0.54_Y_0.15_Cu_0.01_)O_3‐δ_ **|**Ba(Zr_0⋅85_Y_0.15_)O_3‐δ_‐(La_0⋅7_Sr_0⋅3_)V_0.9_O_3‐δ_@CeO_2_‐Pd and (Zr_0.30_Ce_0.54_Y_0.15_Cu_0.01_)O_3‐δ_ has been also used in hydrogen pumping application.
ii. Ba‐based cerate has been used extensively in hydrogen isotope separation applications. Scientists have also improved upon the performance of Nickel doping. Furthermore, Sr‐based cerates have also been used in tritium separation from water decomposition.
iii. Sr‐based cerate conductors with Yb doping are the most widely used PCO for tritium monitoring systems. Most recently, zirconate doping and partial Y doping, and even iron doping have been used for tritium monitoring systems.
iv. Hydrogen sensing applications have been developed using cerate‐based PCO. Y, Zr is used as a doping material for hydrogen sensing applications. Most recently, co‐doping with indium has also been used for its superior stability.

Still, there are several challenges in cerate‐based electrolytes used in electrochemical hydrogen devices. The challenges and research gaps are summarized below:

I. Doping materials, especially rare‐earth elements need to be thoroughly investigated to optimize the properties of the composites; it can increase the overall conductivity and chemical stability for hydrogen pump applications.
II. For hydrogen isotope separation applications, the main challenge is to resolve the high melting point of the proton‐conducting ceramics such as Ba‐cerate or Sr‐cerate. Poor hydrogen flux is also a hindrance in full‐scale implementation. Although scientists are coming up with new ideas to overcome these challenges, a lot of research is still needed.
III. PCO materials suffer from low recovery rate issues when applied in tritium monitoring systems. Also, selectivity and compatibility are also longstanding issues. Aspiring researchers can find ample opportunity in this scope to look for appropriate vacuum conditions for tritium monitoring.
IV. Scientists have made great strides with doping in cerate‐based PCO. However, the study of this doped material has not been done at higher temperatures. Temperature can hugely modulate the hydrogen sensor performance even after doping. The study of temperature dependence is an area where any new scientist can devote their time to make a possible breakthrough.

After reviewing the literature, we have established the following future directions, which provide a clear roadmap for upcoming research:

i. Improve the chemical and mechanical stability of cerate‐based materials by enhancing resistance to CO₂, H₂O, and mechanical stress through composition tuning and structural innovations.
ii. Develop composite and hybrid electrolytes by combining cerates with other conductors or polymers to enhance conductivity, flexibility, and low‐temperature performance.
iii. Advance device integration by optimizing thin‐film cerate electrolytes, interfaces, and multifunctional materials to improve performance and reduce losses.
iv. Ensure scalability and market readiness by using low‐cost, safe materials, proving long‐term reliability, and following standardized testing methods.

## Conflict of Interest

The authors declare no conflict of interest.
